# An Update of Immunohistochemistry in Hepatocellular Carcinoma

**DOI:** 10.3390/diagnostics15172144

**Published:** 2025-08-25

**Authors:** Bingyu Li, Larry Huang, Jialing Huang, Jianhong Li

**Affiliations:** 1 Department of Pathology, Icahn School of Medicine at Mount Sinai-Morningside/West, New York City, NY 10019, USA; 2 College of Arts and Sciences, University of Pennsylvania, Philadelphia, PA 19104, USA; lahuang@sas.upenn.edu; 3 Department of Pathology, Geisinger Medical Center, Danville, PA 17822, USA

**Keywords:** hepatocellular carcinoma (HCC), immunohistochemistry (IHC), diagnostic biomarkers, prognostic markers, predictive biomarkers

## Abstract

Hepatocellular carcinoma (HCC) remains a global health challenge due to molecular heterogeneity and frequent delayed diagnosis. This comprehensive review synthesizes recent immunohistochemistry (IHC) advancements for HCC diagnosis, prognostication, and therapeutic prediction. We systematically evaluate conventional markers, such as hepatocyte paraffin 1 (HepPar1), arginase-1 (Arg-1), and glypican-3 (GPC3), as well as emerging biomarkers, detailing their diagnostic sensitivities and specificities in HCC with varied tumor differentiation. Prognostic immunostaining markers, such as Ki-67 proliferation index and vascular endothelial growth factor (VEGF) overexpression, correlate with reduced 5-year survival, while novel immune checkpoint IHC markers (PD-L1 and CTLA-4) predict response to immunotherapy, particularly in advanced HCC. This work provides evidence-based recommendations for optimizing IHC utilization in clinical practice while identifying knowledge gaps in biomarker validation and standardization.

## 1. Introduction

Globally, primary liver cancer, including HCC and intrahepatic cholangiocarcinoma (ICC), ranks as the sixth most common type of cancer and the fourth leading cause of cancer-related deaths, with approximately 841,080 new cases and 781,631 fatalities reported in 2018 [1]. Projections indicate that by 2025, more than one million people will be diagnosed with liver cancer worldwide annually [2]. HCC, consisting of epithelial cells with hepatocellular differentiation, accounts for 75–85% of all primary liver cancer [3].

Over recent years, the epidemiology of HCC etiology has shifted significantly, with a transition from viral to non-viral causes, especially in regions with high sociodemographic indices [4]. In developed countries, non-alcoholic fatty liver disease (NAFLD, renamed as MASLD (metabolic dysfunction-associated steatotic liver disease [5])) has emerged as the most rapidly growing cause of HCC, with estimates predicting a 56% increase in non-alcoholic steatohepatitis (NASH, renamed as MASH (metabolic dysfunction-associated steatohepatitis [5])) cases over the next decade [6]. Despite advancements in screening and surveillance, HCC is often detected at advanced stages, posing significant challenges for effective treatment and management [7].

Diagnosing and managing HCC presents multiple challenges. First, it is well accepted that early detection significantly improves patient outcomes, but in many developing countries, patients are frequently diagnosed at later stages due to insufficient surveillance [8]. Second, the histologic heterogeneity of HCC and its frequent occurrence alongside chronic liver diseases complicate accurate diagnosis and staging of this liver malignancy [9,10].

Immunohistochemistry (IHC) is a crucial tool for diagnosing HCC. It helps distinguish HCC from other primary liver tumors and liver metastases, ensuring accurate diagnosis for appropriate treatment selection. Immunohistochemical markers such as GPC3, CD34, and HepPar1 have demonstrated high sensitivity in identifying HCC, differentiating HCC from ICC and liver metastasis [11]. IHC technically allows for direct visualization and localization of specific proteins within tissue samples, providing essential immunophenotypic characteristics for accurate classification. In addition, IHC aids in assessing tumor heterogeneity, identifying prognostic factors, and evaluating potential therapeutic targets [12,13]. For instance, recent advancements in IHC techniques have enabled deeper exploration of the tumor microenvironment, including immune checkpoint protein expression across a broad spectrum of malignancies, including HCC [14].

This review provides a thorough overview of recent advancements in diagnostic IHC in HCC. It assesses the latest biomarkers and their diagnostic and prognostic values, highlighting emerging trends in IHC-based clinical research for HCC. By synthesizing recent findings, this review offers pathologists, hepatologists, and oncologists insights into the current landscape and future directions of IHC in HCC management.

## 2. Diagnostic Markers

**Figure 1:**
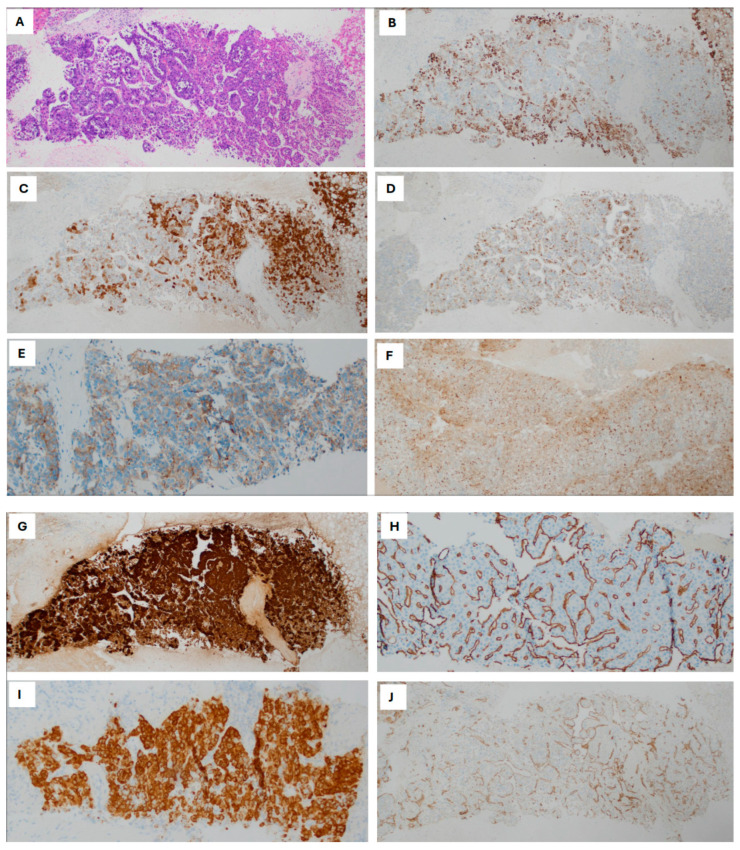
Diagnostic/prognostic markers in HCC. (**A**) HCC, H&E stain; (**B**) HepPar1, cytoplasmic staining; (**C**) arginase-1, cytoplasmic staining; (**D**) glypican-3, predominently cytoplasmic staining; (**E**) Poly-CEA, canalicular staining; (**F**) CD10, canalicular staining; (**G**) GS, cytoplasmic staining; (**H**) CD34, sinusoidal staining; (**I**) albumin ISH, cytoplasmic staining; and (**J**) CD31, sinusoidal staining.

**Figure 2:**
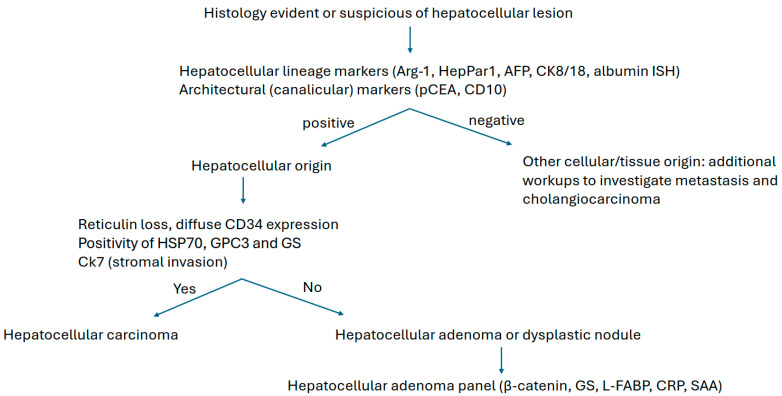
IHC diagnostic pathway for HCC.

Hepatocellular lineage markers include HepPar1 and Arg-1, while canalicular domain markers, such as polyclonal carcinoembryonic antigen (pCEA) and CD10, serve as specific architectural immunomarkers for liver differentiation. Normal hepatocytes express unique cytoskeletal proteins, i.e., cytokeratins (CK) 8 and 18. Notably, aberrant cytokeratin 19 expression in HCC is associated with poor prognosis. Table 1 summarizes currently used diagnostic markers for HCC. Figure 1 illustrates representative HE stains of HCC tissue, accompanied by IHC staining patterns: (A) H&E HCC; (B) HepPar1; (C) Arginase; (D) glypican-3; (E) Poly-CEA; (F) CD10; (G) GS; (H) CD34; (I) albumin ISH; and (J) CD31. Figure 2 illustrates a visual flowchart outlining the IHC diagnostic pathway for HCC.

### 2.1. Classical HCC Markers

#### 2.1.1. Hepatocyte Paraffin 1 (HepPar1)

HepPar1 is a monoclonal antibody that targets the mitochondrial urea cycle enzyme carbamoyl phosphate synthetase 1 (CPS1), which is primarily expressed in hepatocytes. It has been widely recognized in identifying hepatocellular differentiation for its high sensitivity and specificity. The sensitivity of HepPar1 in detecting HCC ranges from 70% to 87%, while the specificity falls between 71% and 97% [15,16,17,18].

HepPar1 typically produces a granular cytoplasmic staining pattern in hepatocytes and HCC cells, making it a valuable tool for distinguishing HCC from metastatic adenocarcinomas and other non-hepatocellular tumors [19,30]. However, HepPar1 is not entirely specific to HCC, as it can stain some non-hepatocellular tumors, particularly those of gastrointestinal origin [29]. Notably, hepatoid tumors, such as those in the gastrointestinal (GI) tract, pancreatobiliary system, lung, and genitourinary tract [46,47], can also stain positive for HepPar1 [48].

Studies have compared HepPar1 to other hepatocellular markers in the diagnosis of hepatocellular lesions. Larson et al. utilized HepPar1 and Arg-1 to differentiate HCCs with cytoplasmic clearing from non-hepatocellular clear cell tumors in liver biopsy specimens. The results indicated that although both HepPar1 and Arg-1 IHCs displayed significantly higher intensity in HCCs than in non-hepatocellular tumors, HepPar1 exhibited lower specificity than Arg-1, as HepPar1 stained a greater proportion of non-hepatocellular tumors compared to Arg-1 [19]. While HepPar1 remains a key marker in the immunohistochemical diagnosis of HCC, its relative lack of specificity urges caution in interpreting the results. This limitation supports the utilization of an IHC panel including more specific hepatocellular markers for accurate diagnosis.

Recent investigations have explored the prognostic value of HepPar1 expression in HCC. A study by Li et al. incorporated HepPar1 staining results into a novel risk-scoring model to predict the early recurrence of HCC after curative resection. Taking HepPar1 with GPC3 and alpha-fetoprotein (AFP) into consideration, this model demonstrated superior prognostic accuracy compared to those using any of these three markers alone [49].

#### 2.1.2. Arginase-1 (Arg-1)

Arg-1 is primarily expressed in normal hepatocytes and plays a crucial role in the final step of the urea cycle. It is currently considered as the best hepatocellular lineage marker. Extensive studies have assessed its diagnostic value in HCC, with most investigations confirming its superiority over other commonly used hepatocellular markers.

In the comprehensive study by Yan et al., Arg-1 exhibited an overall sensitivity of 96.0% in HCC cases, outperforming HepPar1, which showed a sensitivity of 84.1% [15]. This difference was particularly evident in poorly differentiated HCCs, where Arg-1 maintained a sensitivity of 85.7% compared to 46.4% for HepPar1 [15]. Moreover, Arg-1 IHC has shown near-perfect specificity in hepatocellular differentiation.

Elzeftawy et al. conducted a tissue microarray study on 117 cases of liver lesions (77 HCCs, 13 ICCs, and 27 liver metastatic adenocarcinomas with unknown primary). They found that Arg-1 IHC had a 97.5% specificity in distinguishing HCC from ICC and metastatic carcinomas [50]. Arg-1 antibody works better for this differentiation when used in a panel. In a study performed by Wang et al., an IHC panel consisting of Arg-1, GPC3, and HepPar1 achieved an increased diagnostic sensitivity of 89.36% and specificity of 100.00% [20].

Despite its high sensitivity and specificity, the Arg-1 antibody has limitations. There have been rare reports of the negativity of Arg-1 IHC in well-differentiated HCC cases. Krings et al. identified four such cases that still maintained expression of other hepatocellular markers such as HepPar1 and pCEA [21]. Conversely, occasional Arg-1 positivity has been observed in non-HCC tumors. Li et al. reported that 6.90% of ICC cases expressed Arg-1, compared to 76.60% in HCC [20]. Additionally, isolated instances of Arg-1 positivity have been documented in metastatic colorectal and pancreatic cancers [51]. In addition, Arg-1 is frequently positive in hepatoid adenocarcinomas [52], which occur in the GI tract, pancreas, and other sites. 

#### 2.1.3. Glypican-3 (GPC3)

GPC3 is a cell-surface glycoprotein typically expressed during fetal development but is silenced in adult tissues. It regulates cell differentiation and apoptosis. As a heparan sulfate proteoglycan anchored to the cell membrane via glycosylphosphatidylinositol (GPI), GPC3 is overexpressed in 63–92% of HCCs yet is absent in normal adult liver, cirrhotic nodules, and most benign hepatic lesions [31,32,33]. Extensive studies have established the diagnostic utility of GPC3 in HCC, demonstrating an overall diagnostic sensitivity of 75–85% for HCC, which could be maintained as high as 89% in poorly differentiated HCC, and GPC3 outperforms HepPar1 (sensitivity 64%) [31,32].

In a tissue microarray study on 101 cases of HCC (33 well-, 22 moderately, and 12 poorly differentiated), Kaseb et al. reported GPC3 expression rates of 63%, 86%, and 92% for HCCs, respectively [32]. Patients were stratified into clinical score categories (0–3) based on GPC3 staining intensity and the percentage of stained tumor cells in their resection and biopsy specimens. The individuals with GPC3-low expressing tumors (scores 0–1) had a median overall survival of 49.9 months compared to 30.7 months for those carrying tumors of high expressers (scores 2–3), corresponding to a 1.57-fold increase in mortality risk in the latter.

More recent studies revealed the prognostic value of GPC3 expression in HCC. A 2014 meta-analysis by Xiao et al. (n = 1070, across eight studies) indicated that high GPC3 expression correlated with poor overall survival (OS) (HR: 1.96) and disease-free survival (DFS) (HR: 1.99). Subgroup analysis linked strong GPC3 expression to the presence of vascular invasion (OR: 2.43), late TNM stage (OR: 2.26), and high tumor grade (OR: 3.30) [53].

In a meta-analysis by Moudi et al., GPC3 overexpression was associated with a poorer prognosis in patients with hepatitis B-related HCC [54]. A study by Chen et al. on 55 post-hepatectomy patients with early HCC, diagnosed according to the 2009 classifications of the Liver Cancer Study Group of Japan and the International Consensus Group for Hepatocellular Neoplasia, showed that the 5-year DFS rate was significantly lower in patients with GPC3-positive early HCC (27%) than in patients with GPC3-negative early HCC (62%) [55].

Together, these results indicate that GPC3 expression correlates with tumor differentiation and clinical outcomes in HCC.

Recently, efforts have been made to explore the potential of GPC3 as an immunotherapy target in HCC. For example, Zheng et al. conducted clinical trials investigating diverse GPC3-targeted therapy strategies in HCC therapy, including humanized anti-GPC3 cytotoxic antibody therapy, peptide vaccine administration, and immunotoxin treatments [56]. A phase I clinical trial of the anti-GPC3 monoclonal antibodies, codrituzumab combined with atezolizumab, demonstrated not only good tolerance of the agents but also significant suppression of tumor growth in patients with advanced HCC. Among 18 evaluated patients, one case showed partial response (PR), and ten showed stable disease, among whom six were maintained stable for more than 6 months before progression [57].

However, there are limitations of GPC3 in diagnosing HCC. First, its sensitivity can vary among HCC tumor cells with different degrees of differentiation. Shafizadeh et al. observed a drop in the diagnostic sensitivity to 50% in extremely well-differentiated HCC, which mimicked hepatic adenoma [31]. Second, GPC3 expression heterogeneity in the tumor further complicates interpretation, with 15–20% of HCCs demonstrating intratumoral variability in GPC3 expression. The variability is especially pronounced in combined hepatocellular-cholangiocarcinoma tumors, in which the patchy positivity may lead to diagnostic misclassification, particularly in small specimens such as liver core biopsy tissue. Third, focal GPC3 immunoreactivity has been documented in 11% of regenerative nodules in cirrhotic livers, necessitating strict correlation with morphological features to avoid overdiagnosis of well-differentiated HCC [31]. Lastly, while GPC3 exhibits high specificity for HCC, rare cases of positivity have been reported in non-HCC tumors, such as melanoma, nonseminomatous germ cell tumors (e.g., yolk sac tumor, and choriocarcinoma), gastric adenocarcinoma, rare cases of cholangiocarcinoma, neuroblastoma, and lung squamous cell carcinoma [58,59]. While the specificity of GPC3 IHC remains high (94–100%) against metastatic adenocarcinomas [20,60], 14–21% of ICC cases showed focal GPC3 staining [61,62], which necessitated additional IHC markers (e.g., CK19, mucin) for further differentiation [22,33]. Li et al. reported GPC3 expression in 5.3% of liver metastatic carcinomas and emphasized prudential interpretation of GPC3 IHC results in conjunction with clinical and histopathological findings [63].

#### 2.1.4. Polyclonal Carcinoembryonic Antigen (pCEA)

Carcinoembryonic antigen (CEA) is a glycoprotein primarily expressed in fetal epithelial cells and, to a lesser extent, in normal adult tissues. The utility of pCEA IHC in diagnosing hepatocellular tumors takes advantage of the cross-reactivity of this antibody to biliary glycoproteins to yield a distinctive canalicular staining pattern. This pattern results from the localization of pCEA along bile canaliculi, setting it apart from the cytoplasmic or membranous staining commonly seen in adenocarcinomas. This unique staining characteristic is specific to hepatocellular differentiation [64,65,66].

The canalicular staining pattern of pCEA is highly specific to HCC and observed in 60% to 90% of cases, particularly in well- and moderately differentiated tumors [64,66]. In contrast, liver metastatic adenocarcinomas and cholangiocarcinomas typically display diffuse cytoplasmic or membranous staining, making pCEA a valuable marker for distinguishing primary liver tumors from metastatic lesions [66]. However, the sensitivity of pCEA declines significantly in poorly differentiated HCC compared to well- and moderately differentiated HCC ones, as the canalicular staining pattern may be lost or replaced by diffuse cytoplasmic staining.

Immunostaining for pCEA is commonly used as part of an IHC panel alongside markers, such as HepPar1, CD10, and Arg-1, to confirm hepatocellular differentiation and malignancy. This approach is particularly useful when histological features are unclear or only a limited biopsy sample is available [67,68]. While the canalicular staining pattern is specific to HCC, interpretation of pCEA IHC can be challenging due to technical variations, background staining, and overlapping patterns with other tumor types. For example, intense canalicular staining may resemble the membranous staining observed in liver metastatic adenocarcinomas, necessitating careful evaluation [69].

#### 2.1.5. CD10

CD10 (neutral endopeptidase) is a cell surface metalloproteinase that plays a critical role in hepatocytic differentiation through its enzymatic activity in degrading bioactive peptides, including the inactivation of hepatocyte growth factor and other bioactive peptides that influence cellular proliferation and differentiation [66,70]. In HCC, CD10 demonstrates a unique canalicular staining pattern due to its localization on bile canalicular membranes, reflecting preserved hepatocytic polarization. This immunohistochemical feature has been extensively studied for diagnostic applications in liver pathology, particularly for distinguishing HCC from metastatic carcinomas and benign hepatic lesions [70].

CD10 IHC exhibits moderate sensitivity but high specificity for determining hepatocellular differentiation, particularly displaying a canalicular staining pattern. In a seminal study of 55 liver FNAB (fine-needle aspiration biopsy) cases (22 HCCs, 23 metastases, and 10 benign), Lin et al. found an average diagnostic sensitivity of 86% (19/22 HCC cases) across all HCC cases with canalicular expression of CD10, with sensitivities of 82% in well-differentiated HCCs and 91% in moderately to poorly differentiated HCCs [27]. In the same study, the diagnostic specificity reached 87%, though renal cell carcinoma and lung adenocarcinoma metastasizing to the liver showed cytoplasmic/membranous staining [27]. These findings were corroborated in a study of CD10 IHC in 50 liver lesions (25 HCCs and 25 metastases) demonstrating a diagnostic sensitivity of 68% (17/25 HCC cases) and a specificity of 84%, with nonspecific cytoplasmic or membranous staining in four cases of metastatic carcinoma (one pancreatic primary, two gallbladder primary, and one of unknown primary) [71].

Recent studies shed new light on the role of CD10 IHC in diagnosing liver lesions. Wen et al. evaluated CD10 expression in 136 hepatic tumors (105 HCCs, 12 focal nodular hyperplasia [FNH], and 19 ICCs) and found CD10 expression in 61% of HCCs, 100% of FNH, and 31.6% of ICC tumors [28]. The study also detailed different CD10 expression patterns in HCC, including cell membrane (13/64; 20.3%), luminal (9/64; 14.0%), cytoplasmic puncta (15/64; 23.4%), and canalicular (27/64; 42.3%) patterns. Membranous CD10 staining correlated with steatotic changes in HCC, while canalicular pattern predominated in both FNHs (100%) and HCCs [28]. Furthermore, the authors reported CD10 expression in tumor cells of 31.6% (6/19) ICC cases, including one case of cell membrane, three cytoplasmic, and two luminal staining patterns.

The drawbacks of CD10 IHC in distinguishing liver lesions are worth mentioning. CD10 does not differentiate malignant and benign hepatocytes since they all theoretically express CD10 [27]. Also, ICC tumor cells may express this marker [28].

#### 2.1.6. Cytokeratin 8 and 18

Cytokeratin 8 and 18 (CK8/18) are intermediate filament proteins constitutively expressed in normal hepatocytes and play key roles in maintaining cell architecture and signaling homeostasis. CK8/18 is expressed in normal and neoplastic hepatocytes. CK8/18 overexpression in HCC is mechanistically linked to neoplastic transformation through phosphorylation-dependent complex formation [72]. Loss of CK8/18 represses the activation of focal adhesion kinase and promotes the metastatic potential of HCC cells [25].

CK8/18 IHC exhibits high sensitivity but variable specificity for HCC detection, depending on tumor differentiation and background liver pathology. In a study of 53 liver biopsies, CK8/CK18 positivity was observed in 83.3% and 83.3% of HCCs versus 45.5% and 36.4% in chronic active hepatitis B, 20% and 26.7% in chronic active hepatitis C, and 90% and 70% in autoimmune hepatitis (AIH), respectively. A retrospective study of 42 HCC cases reported CK8 and CK18 positivity in 54.54% and 75.75% of tumors, respectively, with stronger expression in well-differentiated HCCs compared to poorly differentiated tumors [26].

Serum CK18 may serve as a diagnostic marker for HCC workup, as suggested by multiple lines of data. In a study with 90 Egyptian subjects (30 with cirrhosis, 30 with HCC, and 30 healthy volunteers), serum CK18 levels were significantly elevated in HCC cases (1247.8 ± 105.3 U/L) than those in cirrhotic patients (834.1 ± 38.8 U/L) and healthy volunteers (265.2 ± 83.1 U/L), with a diagnostic sensitivity of 95.6% and specificity of 93.3% for HCC detection. When combined with serum AFP quantification, the diagnostic sensitivity reached 98% [73].

Serum-fragmented CK18 (fCK18) levels, which reflect overall liver function, the degree of liver fibrosis, and the progression of HCC, could be used as a potential predictor of survival in HCC patients. In a study of 497 chronic liver disease patients (297 outpatients and 200 hospitalized with HCC), serum-fragmented CK18 (fCK18) level < 1.15 ng/mL was identified as an independent predictor of survival (HR = 3.5) [74].

Similar to other IHC markers for a diagnosis of HCC, CK8/18 expression is differentiation-dependent, and the expression variability reduces the sensitivity of CK8/18 IHC in recognizing poorly differentiated HCCs [26].

#### 2.1.7. CK19

Cytokeratin 19 (CK19) is a cytoskeletal structural protein. In the liver, it is typically expressed in biliary epithelium and hepatic progenitor cells. Its expression in HCCs usually correlates with aggressive histopathology and poor clinical outcomes.

In a study of 120 HBV-related HCC patients, CK19 IHC positivity (15%) was associated with higher serum AFP levels, advanced peritumoral desmoplastic reaction, and proliferative peritumoral ductular reaction [44]. Another smaller study with 24 HCC patients by Su et al. found that tumor-free survival was significantly shorter in the CK19+ primary HCC group than that in the CK19− group (3 vs. 27 months) [75]. A similar conclusion was drawn in a study of 206 HCC patients who underwent liver transplantation, linking CK19 expression to a 2.5-fold higher recurrence risk and possible resistance to sorafenib [76]. These results also implied that CK19 could potentially predict the therapeutic benefit of regorafenib, though this point needs to be validated in large, multicentric clinical trials [76].

A recent study explored the preoperative prediction of CK19 expression by imaging. The study retrospectively enrolled 158 treatment-naïve patients with solitary HCC who underwent curative resection and preoperative gadoxetic acid–enhanced MRI with T1 mapping within two weeks before surgery. Patients from Institution I (n = 102) formed the training cohort, and those from Institution II (n = 56) served as an external test cohort. Imaging analysis was used to measure T1 relaxation times on unenhanced and 20 min hepatobiliary phases, relative ADC (rADC), and semantic features (e.g., margin and target sign). Multivariable logistic regression identified AFP > 400 ng/mL (OR 4.6), rADC ≤ 0.71 (OR 3.5), and hepatobiliary-phase T1 relaxation time > 797 ms (OR 4.5) as independent predictors of CK19 expression. The proposed model achieved a C index of 0.844 in the training cohort and 0.818 in the external test cohort, indicating robust efficacy in predicting CK19 expression [77].

A multicenter study of 141 surgically resected HCCs demonstrated an AUC of 0.82 for predicting CK19 expression by IHC with a deep learning radiomics (DLR) model based on gadoxetic acid–enhanced MRI hepatobiliary phase (HBP) images. The sensitivity and specificity for predicting CK19 expression with this model reached 80% and 76.6%, respectively, with the sensitivity improved to 96% when the serum AFP level of >400 ng/mL was integrated into the model [45].

### 2.2. Novel Diagnostic Markers

#### 2.2.1. Glutamine Synthetase (GS)

GS, an enzyme involved in nitrogen metabolism and a key target of the β-catenin pathway, exhibits distinct expression patterns in HCC. It catalyzes the conversion of glutamate and ammonia into glutamine within the liver. In a normal liver, GS expression is confined to perivenular hepatocytes, but in HCC, it often presents with diffuse and homogeneous staining in more than 50% of tumor cells [39,78].

GS IHC serves as a valuable tool in distinguishing between benign and malignant hepatocellular lesions. Factually, GS positivity possesses a diagnostic sensitivity of 43.9–100% towards HCC in cirrhotic livers [36]. Differentiating low-grade liver neoplasms is challenging radiologically and pathologically, but GS IHC can be especially useful for this purpose. Lagana et al. constructed a tissue microarray based on 30 low-grade HCC (LG-HCC) and 18 hepatocellular adenoma (HCA) cases, and defined GS positivity as at least 50% of tumor cells demonstrating immunoreactivity. This study yielded a sensitivity of 80% and a specificity of 50% for distinguishing LG-HCC from HCA [37].

In a multicenter study of 260 liver tissue samples (120 HCC, 90 chronic hepatitis B [CHB] in stage 4, and 50 CHB in stage 1–3), Long et al. found a GS IHC positive rate of 70% in HCCs, compared to 46.7% in stage 4 CHB and 38% in stage 1–3 CHB [79]. A study by Moudi et al. included a total of 121 cases, comprised of patients with HBV infection alone (40), HCC without HBV (41), and HBV-related HCCs (40) for primarily assessing the utility of GS IHC in diagnosing early HCC, using 30 normal liver tissue samples as controls [80]. They reported a sensitivity of 60.7% and specificity of 94.3% for GS IHC in differentiating HCC from other hepatic lesions due to hepatitis B virus (HBV) infection [80].

The synergistic application of GS with another marker, such as heat shock protein 70 (HSP70) and GPC3, has emerged as a robust diagnostic strategy for distinguishing HCC from preneoplastic lesions and benign hepatocellular proliferations. A multicenter study by Di Tommaso et al. analyzed 176 biopsies encompassing large regenerative nodules (n = 13), low-grade dysplastic nodules (n = 21), high-grade dysplastic nodules (HGDN; n = 50), and HCCs stratified by differentiation (very well-differentiated (VWD, n = 17; well-differentiated (WD, grade 1), n = 40; moderately/poorly differentiated (MPD, n = 35) [81]. The panel (HSP70, GPC3, and GS) demonstrated 100% specificity for HCC detection when ≥2 markers were positive, with sensitivity increasing from 23.5% in VWD-HCC to 74% in MPD-HCC [81]. Notably, no HGDN cases exhibited dual positivity, while 72.9% of WD-G1/VWD-HCC cases showed ≥2 markers [81].

Recent research suggested a potential prognostic value of GS expression in HCC. Shao et al. conducted a retrospective analysis of 431 HCC patients who underwent curative hepatectomy between 2010 and 2019 at West China Hospital. Among them, 251 patients received hepatectomy alone, while 180 received sorafenib as adjuvant treatment after surgery. In the hepatectomy-only group, GS-negative patients showed significantly better overall survival (OS) and recurrence-free survival rates (RFS) compared to GS-positive patients. The median RFS was 52.0 months for GS-negative patients compared to 16.0 months for GS-positive patients. Among patients who received adjuvant sorafenib, those with GS-negative tumors demonstrated a significantly better response to the treatment. The 1-year and 3-year RFS rates for GS-negative patients in the sorafenib group were 89.9% and 71.7%, respectively, compared to 78.2% and 60.9% in the control group [38]. The findings suggest that GS expression status could serve as a simple and applicable approach for predicting patient prognosis and guiding targeted therapy in HCC [38].

GS IHC has notable pitfalls in identifying HCC. This marker is expressed in 27–46.7% of non-malignant liver diseases, including CHB and cirrhosis [79]. Diffuse GS positivity (≥50% tumor cells) is specific for HCC, but focal or heterogeneous patterns occur in 10–23% of HGDN cases [78].

#### 2.2.2. Heat Shock Protein 70 (HSP70)

HSP70, a member of the heat shock protein family, plays a crucial role in protein folding, cellular stress response, and cytoprotection. In the context of HCC, HSP70 has gained considerable attention due to its overexpression in malignant hepatocytes. This overexpression is believed to be a response to the persistent stress conditions within the tumor microenvironment, enabling cancer cells to adapt and survive [82].

HSP70 is a molecular chaperone that facilitates the folding of newly synthesized proteins and the refolding of damaged proteins. In neoplastic conditions, it facilitates tumor survival by suppressing apoptosis and promoting proliferation by inhibiting mitochondrial permeability transition pores to prevent cytochrome c release and caspase activation [83]. HSP70 expression is upregulated in HCC, contributing to tumor cell survival and resistance to apoptosis [84].

In a study of 56 primary hepatic lesions (5 FNHs, 5 hepatic adenomas, 33 cases of small, vaguely nodular lesions (5 dysplastic nodules, 28 early HCCs), and 13 late HCCs), HSP70 IHC exhibited a sensitivity of 78.2% and a specificity of 100% for HCC detection when moderate-to-strong nuclear staining in ≥10% of tumor cells was considered as positive [85], reinforcing its role as a reliable biomarker in recognizing malignant hepatocytes. HSP70 is also helpful for diagnosing very early hepatocellular carcinoma. In a prospective study evaluating expression of HSP70 alongside GPC3 and GS in 60 cirrhotic patients with ultrasound-detected nodules measuring 5–20 mm (40 confirmed HCCs and 20 controls with non-malignant nodules), HSP70 exhibited a sensitivity of 57.5% and a specificity of 85%, when positive staining was defined by crisp nuclear/cytoplasmic expression in neoplastic cells [39]. However, the combination of HSP70 with GPC3 IHCs achieved perfect specificity (100%), though unsatisfactory sensitivity (40%) [39].

HSP70 is currently considered a valuable immunohistochemical marker for differentiating WD-HCC. Nguyen et al. evaluated HSP70 expression in a cohort of 107 hepatocellular lesions, including 17 typical hepatocellular adenomas, 20 atypical hepatocellular neoplasms (14 clinically atypical and 6 pathologically atypical), 13 very well-differentiated HCC, 43 well-differentiated HCC (20 in non-cirrhotic liver and 23 in cirrhotic liver), and 15 HGDNs [86]. The HSP70 expression was observed in 68% of HCCs, with similar positivity rates in very well-differentiated HCC (71%) and well-differentiated HCC (67%) [86]. Importantly, HSP70 was negative in all typical hepatocellular adenomas, yet positive in only 10% of atypical hepatocellular neoplasms and positive in just 6% of HGDNs. Other investigators also explored the utility of combined HSP70 and GS IHCs, finding that dual positivity was present in 45% of all HCCs, compared to only 10% of atypical hepatocellular neoplasms, but none in the typical adenomas [86]. Several studies found that a panel of three IHC markers (GPC3, GS, and HSP70) is useful for detecting well-differentiated HCC in biopsies [87,88].

HSP70 expression has considerable prognostic relevance in HCC. Joo et al. conducted a comprehensive study examining HSP70 expression in 71 surgically resected HCC cases [40]. Their cohort included tumors of varying differentiation based on Edmondson–Steiner grading (20 well-differentiated and 51 poorly differentiated) and diverse etiologies (52 HBV-associated, 7 HCV-associated, and 12 non-viral cases). Using monoclonal antibodies and a three-tiered scoring system (<10% negative, 10–50% 1+, and >50% 2+), they found HSP70 immunoreactivity in 56.3% of HCCs, with both cytoplasmic and nuclear staining displaying a characteristic fine granular pattern. They demonstrated significant correlations between HSP70 expression and adverse prognostic factors, including large tumor size (*p* = 0.0129), presence of portal vein invasion (*p* = 0.0231), high tumor stage (*p* = 0.0392), and higher Ki-67 labeling indices (*p* = 0.0159). While in sarcomatoid HCCs (n = 63), Zhou et al. found that HSP70 predicted OS and RFS when used in a prognostic panel including GS and GPC3 [89].

The pitfalls of HSP70 IHC are noticeable. On one hand, HSP70 expression is not exclusive to HCC, as liver metastatic adenocarcinomas and cholangiocarcinomas may show immunoreactivity [37]. On the other hand, tumor heterogeneity further complicates interpretation, particularly in poorly differentiated HCCs, where HSP70 expression may diminish [40].

#### 2.2.3. CD34

As a transmembrane phosphoglycoprotein, CD34 is primarily expressed on hematopoietic stem cells and endothelial cells. The process of sinusoidal capillarization represents a fundamental morphological alteration during hepatocarcinogenesis, characterized by phenotypic changes in liver sinusoidal endothelial cells (LSECs).

In normal liver parenchyma, hepatic sinusoids lack conventional basement membranes and exhibit distinctive fenestrations. During malignant transformation, these sinusoids progressively acquire features of continuous capillaries, including the formation of basement membranes and loss of characteristic fenestrations. This architectural remodeling significantly impacts hepatic hemodynamics and contributes to the altered microenvironment that supports tumor growth and progression. The expression of CD34, a marker typically absent in normal liver sinusoids, emerges as a reliable indicator of this capillarization process. In normal liver tissue, its expression is restricted to portal vessels and a few periportal sinusoids. However, as liver disease progresses toward HCC, CD34 expression increases significantly, reflecting the transformation of sinusoidal endothelial cells into continuous capillaries [42,90].

The transition from normal liver to HCC is accompanied by progressive changes in CD34 expression patterns. In chronic hepatitis and early cirrhosis, sinusoidal CD34 immunoreactivity begins to emerge focally, particularly in periportal areas and around cirrhotic nodules, though staining remains sparse with weak to moderate (+/++) intensity [91]. As lesions progress to dysplastic nodules, particularly high-grade dysplasia, CD34 staining becomes more pronounced but typically maintains a peripheral or focal distribution [43]. Upon transformation of dysplastic nodules to HCC, CD34 expression undergoes dramatic amplification, with diffuse and strong (+++) sinusoidal staining becoming evident in more than 50% of sinusoidal cells regardless of tumor grade [43].

Recent studies have demonstrated the high sensitivity of CD34 immunostaining in diagnosing HCC. A retrospective study from Northeast India found that 92.8% of HCC tumors exhibited strong, complete, and diffuse CD34 expression in sinusoidal vessels in tumors with a hepatic plate thickness of more than three cell plates. In contrast, benign liver conditions typically showed either negative or weak CD34 expression in hepatic sinusoids, with periportal and perinodular staining observed in only 35.1% of cases [92].

Reticulin loss in HCC disrupts the normal trabecular architecture, while CD34 highlights the aberrant vascular network; thus, the integration of CD34 IHC with reticulin staining can enhance diagnostic accuracy. In a landmark study, comprised of 65 HCC cases, 29 non-malignant neoplastic nodules (FNH, n = 10; large regenerative nodules, n = 6; low-grade dysplastic nodules, n = 3; HGDN, n = 7; and liver cell adenomas, n = 3) and 30 hepatolithiasis (as controls), HCCs exhibited diffuse CD34 positivity in >50% of sinusoidal cells (62/65 cases, 95.4%) alongside abnormal reticulin patterns characterized by thin, incomplete networks surrounding expanded trabeculae (>3 cell layers) [42]. In contrast, non-malignant lesions showed preserved reticulin architecture (72.4% specificity) and limited CD34 expression (<20% sinusoidal staining). The combined reticulin+/CD34+ panel achieved a sensitivity of 95.4% and a specificity of 86.2% for HCC diagnosis, outperforming individual markers [42].

Recent exploration of the relationship between the expression of CD34 and other molecular markers in HCC revealed an inverse correlation between the expression of CD34 and C-type Lectin Domain Family 4 Member G (CLEC4G) in HCC tumor tissues. CLEC4G, typically expressed in liver sinusoidal endothelial cells, decreased, whereas CD34 expression increased as HCC progressed, supporting the notion of sinusoidal capillarization in HCC [90].

While CD34 shows significant promise as a diagnostic marker for HCC, it is crucial to interpret its expression in conjunction with other clinical and pathological findings, as the sensitivity and specificity of CD34 alone may not be sufficient for a definitive diagnosis, particularly in well-differentiated or early-stage HCC. Therefore, using CD34, in combination with markers such as Golgi protein-73 (GP73) and GPC3 as a diagnostic panel, is recommended to enhance diagnostic accuracy in challenging cases [42].

#### 2.2.4. Albumin mRNA In Situ Hybridization (ISH)

Albumin ISH is an extremely useful tool in diagnosing lesions of hepatobiliary primary. This detection pictures the presence and location of albumin mRNA within cells in tissue sections using RNA-targeted probes designed to bind specifically to albumin mRNA. This circumvents the limitations of traditional IHC, which often carries nonspecific background staining due to unavoidable contamination of serum albumin in the tested tissue. Albumin ISH is a sensitive and specific method for identifying lesions of hepatocellular or small bile duct origin. Applying signal amplification technology can improve diagnostic sensitivity. For example, platforms such as RNAscope™ (ACD Bio) employ a double-Z probe design to enhance signal-to-noise ratios, while branched-chain RNA ISH (e.g., Affymetrix) amplifies RNA signals through sequential hybridization steps, to achieve high sensitivity and specificity [22].

Albumin ISH has demonstrated good diagnostic performance in identifying HCC, even in poorly differentiated cases. Shahid et al. examined 93 HCCs (6 well-differentiated, 51 moderately differentiated, and 36 poorly differentiated) along with an extensive range of potential mimics, including neuroendocrine tumors of the gastrointestinal tract (n = 31), pancreatic neuroendocrine tumors (n = 163), melanoma (n = 15), and gallbladder carcinoma (n = 34) [22]. Albumin ISH demonstrated 99% sensitivity for HCC, with 92 of 93 cases exhibiting positive staining, significantly exceeding the performance of traditional immunohistochemical markers, HepPar1 and Arg-1, which showed sensitivities of 84% and 83%, respectively. Furthermore, 97% of HCCs showed albumin positivity in >50% of tumor cells, compared to only 76% for HepPar1 and 70% for Arg-1. The single HCC case that was negative on albumin ISH was a poorly differentiated tumor with suboptimal mRNA preservation related to prior radiofrequency ablation. Albumin ISH performed exceptionally well in poorly differentiated HCC, where traditional markers often underperform: Albumin ISH maintained a sensitivity of 99% in poorly differentiated HCCs, significantly surmounted both Arg-1 (71%) and HepPar1 (64%) [22]. Ferrone et al. conducted a large study involving 42 HCCs and 83 ICCs, along with 332 non-hepatic carcinomas from various sites, including lung, breast, pancreas, stomach, and colon. They reported albumin ISH positivity in 100% of HCCs and 99% of ICCs, confirming its high sensitivity for liver-origin tumors [23].

The specificity of albumin ISH is nuanced. Nasir et al. found that albumin ISH demonstrates high sensitivity for hepatocellular origin, detecting 100% of HCCs (22 conventional HCCs and 4 fibrolamellar carcinomas) and 81% of ICCs (22/27 cases) in a multicenter study. While albumin ISH was highly sensitive for cancers of hepatocellular origin, they also noted focal positivity in a subset of non-hepatocellular tumors: 39% of gallbladder adenocarcinomas (5/13 cases evaluated), 20% of lung adenocarcinomas (3/15 cases), 25% of yolk sac tumor (2/8 cases evaluated), 29% of acinar cell carcinoma (2/7 cases evaluated), hepatoid pancreatic adenocarcinoma (n = 1 of 1), and 18% of breast invasive ductal carcinomas (2/11 cases) [24]. Notably, these non-hepatocellular tumors displayed patchy staining, distinct from the diffuse expression pattern in HCC and ICC [24].

Recent studies have highlighted evolving insights into the oncogenic role of albumin in the development of HCC. Albumin protein acts as a tumor suppressor and plays a key role in HCC progression, particularly in tumor invasion and metastasis [93]. Reduced albumin expression correlates with aggressive tumor behavior, potentially through downregulating metastasis-associated genes such as urokinase plasminogen activator surface receptor (uPAR) and matrix metalloproteinase (MMP) [93].

We conducted a small-scale investigation of IHC markers of HCC at the Geisinger Medical Laboratories (GML). On examination of 18 HCC, as shown in Table 2 [94], our results demonstrated comparability to those in the literature, recapitulating the reliability and reproducibility of these immunohistochemical markers in routine clinical practice.

The diagnostic accuracy and prognostic utility of IHC markers in HCC varies significantly across histological subtypes, disease stages, and etiological backgrounds, necessitating subtype-specific diagnostic approaches and prognostic stratification strategies. Conventional HCC markers exhibit notable limitations in specific HCC variants. Here are some examples: Scirrhous HCC has significantly reduced HepPar1 positivity compared to conventional HCC (26% vs. 74%) while frequently expressing adenocarcinoma-associated markers, including CK7 (53% vs. 2%), CK19 (26% vs. 2%), and epithelial cell adhesion molecule (EpCAM, 63% vs. 11%) [95]. Fibrolamellar HCC possess distinctive immunophenotypic characteristics featuring uniform CK7 and epithelial membrane antigen (EMA) positivity (100% vs. <33% in conventional HCC), while maintaining expression of hepatocellular markers including HepPar1 (sensitivity of 78% vs. 85%, compared to conventional HCC) and variable GPC3 expression (59% vs. 39% in conventional HCC) [96]. Clear cell HCC needs to be distinguished from metastatic renal cell carcinoma – it stains positive for HepPar1 in 82–97% and albumin ISH in 100% of cases, but negative for EMA and LeuM1 [97]. Sarcomatoid HCC displays a complex immunophenotype characterized by the retention of cytokeratin and vimentin expression but complete loss of hepatocellular markers, including HepPar1 and arginase-1, in sarcomatoid areas [98,99]. Etiological differences in HCC also significantly influence the performance of diagnostic markers. HCCs due to viral infection (particularly HBV-related) show distinct transcriptomic profiles and immune infiltration patterns compared to non-viral ones, potentially affecting biomarker expression patterns and necessitating etiology-specific diagnostic algorithms [100,101]. Geographic variations further complicate the utility of HCC markers, as geographic differences in viral etiology prevalence (93% HBV-related HCC in China vs. higher HCV prevalence in Western populations) may affect population-specific diagnostic accuracy of IHC panels [102]. These findings underscore the critical need for subtype-specific validation of immunohistochemical markers, integration of molecular diagnostic approaches, and development of etiology-stratified diagnostic algorithms that take into consideration the distinct biological characteristics and marker performance variability across different HCC variants and patient populations [103].

## 3. Prognostic Markers

### 3.1. Cell Cycle Regulators

#### 3.1.1. p53

The p53 IHC staining has been used for a long time in diagnosing and assessing the prognosis of various malignancies. Its diagnostic judgment is based on the accumulation of mutant p53 protein or complete loss of p53 protein in tumor cells, also known as the mutant or null phenotype, respectively. In normal cells, wild-type p53 has a short half-life with an average half-life of just nine minutes [104] and is typically undetectable by IHC. However, mutations in the *TP53* gene, which are common in HCC, produce mutant p53 proteins that are resistant to Mdm2-mediated degradation, causing the buildup of the stable mutant p53 protein in the nucleus, making it visible on IHC [105]. 

*TP53* is the most critical tumor suppressor in the human body, so loss of the function of the p53 protein usually predicts poor prognosis in most malignancies. Therefore, p53 staining is often used as a valuable marker for risk assessment and treatment planning in many malignancies, including HCC.

The 2016 meta-analysis by Liu et al. evaluated 36 studies with a total of 1659 HCC patients. This analysis compared p53 IHC overexpression (using antibodies such as PAb1801 and DO-7) against *TP53* mutation status. The study showed a sensitivity of 83% and a specificity of 74% for detecting *TP53* mutations by p53 IHC. More specifically, the PAb1801 antibody outperformed other prognostic markers. However, cross-study heterogeneity in IHC protocols and cutoffs (e.g., 10% vs. 20% positivity thresholds) contributed to varied positivity rates reported across regions, ranging from 22.8% in U.S. studies to 91% in Romanian cohorts.

A 2023 cross-sectional study of 41 surgically resected HCC cases revealed p53 expression in 35 patients (85.4%) of tumors (8 weak, 11 moderate, 16 strong expression) using nuclear staining thresholds ≥ 10% positivity [106]. This cohort included diverse histological subtypes of HCC: 17 classic HCC, 11 steatohepatitic, 9 macrotrabecular-massive, and 4 clear cell variants. The study utilized non-neoplastic liver tissue as controls and found no significant correlation of p53 expression with AJCC stages (IB-IIIA) or specific subtypes. In the HCC cohort, p53 expression was significantly associated with tumor differentiation status, with higher levels in poorly differentiated tumors (40.40 ± 25.697% expression rate) compared to well-differentiated counterparts. A higher percentage of positive p53 expression was observed in male patients over 60 years old, particularly in those with single HCC nodules exceeding 5 cm in diameter and vascular invasion.

#### 3.1.2. Ki-67

Ki-67 is a widely used IHC marker to assess cell proliferation in different types of tumors. The Ki-67 antibody reacts with the antigen expressed in all the cell cycle phases (G1, S, G2, and M) but not in quiescent cells (G0) [107]. This temporal expression pattern of Ki-67 protein allows for quantifying tumor proliferative activity by Ki-67 staining, with the labeling index (LI) typically reported as the percentage of positively stained nuclei among total tumor cells examined [41].

Clinical studies have demonstrated the prognostic value of Ki-67 LI in HCC management. Patients with a HCC of Ki-67 LI > 10% had significantly shorter 5-year DFS and OS, compared to Ki-67 low-expressing cases, regardless of TNM stage [41]. A meta-analysis of 54 studies (n = 4996) found similar associations, showing that high Ki-67 LI correlated with advanced histological grade (OR = 3.21), vascular invasion (OR = 2.89), and metastatic disease (OR = 2.45) [108]. A recent research in transplanted populations revealed similar trends, with Ki-67 LI > 5% predicting worse RFS (HR = 2.1) and OS (HR = 1.8) among 114 HCC patients [109].

The role of Ki-67 as a proliferation marker in HCC has been reinforced by recent transcriptomic analyses, which demonstrated that high Ki-67 gene expression (MKi67) correlates with histologic progression from cirrhotic liver to advanced HCC, independent of tumor grade [110]. Ramos-Santillan et al. analyzed transcriptomic data from 358 HCC patients in the Cancer Genome Atlas (TCGA) cohort and 115 patients in the Gene Expression Omnibus (GSE76427) validation cohort [110]. Patients were stratified into MKi67-high and MKi67-low groups based on median gene expression levels. Notably, MKi67 expression increased incrementally across the carcinogenic sequence, with the lowest levels in normal liver and the highest in advanced HCC. MKi67 expression in cirrhotic liver was comparable to that in normal liver but increased in early HCC and further in advanced HCC. Critically, this association remained significant after adjusting for tumor grade. Subgroup analyses of TCGA data revealed that MKi67-high tumors had worse DFS, disease-specific survival (DSS), and OS across all histologic grades (G1–G3), with hazard ratios exceeding 5.42 for high-risk patients. Even in grade 1 tumors, MKi67-high cases exhibited a 3.5-fold increased risk of recurrence compared to those with MKi67-low expression. The study’s multivariate Cox regression analysis identified MKi67 as an independent prognostic factor for HCC development in cirrhotic patients (HR = 5.42). These results underscore Ki-67’s role not only as a histologic progression marker but also as a predictor of aggressive behavior in HCC, independent of conventional grading systems [110].

Adequate caution should be exercised in the interpretation of Ki-67 IHC results for several reasons. Interstudy variability in staining protocols and LI cutoff values (ranging from 5% to 50%) [41,108,109,111] complicates cross-trial comparisons and clinical application. Additionally, appropriate controls are essential but inconsistently applied [41,112]. Pathophysiologically, chronic liver disease itself alters hepatocellular proliferative activity, so control selection must account for underlying liver pathology. Furthermore, standardization of Ki-67 LI quantification adds another layer of complexity in the evaluation of proliferation activity.

### 3.2. Angiogenesis-Related Markers: Vascular Endothelial Growth Factor (VEGF) and CD31

#### 3.2.1. Vascular Endothelial Growth Factor (VEGF)

Angiogenesis plays a crucial role in the progression and development of HCC. Numerous angiogenesis-related markers have been studied for their potential role in the diagnosis, prognosis, and treatment of HCC, with VEGF being one of the most extensively researched. VEGF is upregulated in HCC due to hypoxia-driven mechanisms involving hypoxia-inducible factors (HIF-1α and HIF-2α), which promote tumor vascularization and progression [113]. High serum VEGF expression levels are associated with poor prognosis in HCC. Histologically, VEGF expression is often significantly higher in HCC tissues compared to normal liver tissue, reflecting the increased angiogenic activity within tumors [114,115].

Clinically, VEGF IHC may serve as a prognostic marker. In a study of 105 HCC patients, 68.6% of tumors demonstrated VEGF-positive staining in the tumor tissues. Capsular infiltration, vascular invasion, and intrahepatic metastasis were observed more frequently in VEGF-positive tumors than in VEGF-negative ones [116].

Another study with 234 patients with HCCs following surgical resection showed that VEGF expression was an independent prognostic factor for overall survival [117]. In this comprehensive analysis, a standardized immunohistochemical scoring system was employed, stratifying VEGF expression into low [VEGF (−)/(+)] and high [VEGF (++)/(+++)] categories based on staining intensity and percentage of positive cells [117]. Multivariate analysis revealed that a high level of VEGF expression in tumor tissue (≥50% positive cells) was associated with reduced 5-year OS (54.3% vs. 82.6% in low-expression groups), and the VEGF expression and tumor T stage were independent prognostic factors for overall survival (HR = 2.573 and 4.953, respectively) [117].

Comparably, serum VEGF levels > 500 pg/mL were an independent preoperative factor predictive of microscopic venous invasion in 100 resected HCC cases [113]. In a median duration of follow-up of 11.6 months, serum VEGF levels > 500 pg/mL were associated with a higher postoperative recurrence (48% vs. 27%) [113].

The correlation between VEGF overexpression and poor prognosis in HCC appears to be due to VEGF-mediated angiogenesis and potential enhancement of tumor invasiveness, which could be a therapeutic target in HCC.

VEGF-targeted therapies in HCC have illustrated remarkable clinical benefit across multiple therapeutic lines. The IMbrave150 phase III trial disclosed an improved median overall survival in patients with unresectable HCC from 13.4 months with therapy of sorafenib to 19.2 months when treated with the combinatory therapy of bevacizumab (anti-VEGF antibody) and atezolizumab (PD-L1 inhibitor) [118].

In the second-line therapy setting, the REACH-2 trial validated ramucirumab (VEGF receptor-2 inhibitor) as an effective therapy for patients with advanced HCC and elevated AFP ≥ 400 ng/mL, with improved median overall survival of 8.5 months versus 7.3 months with placebo [119]. Additionally, combination approaches incorporating VEGF inhibition, such as transarterial chemoembolization (TACE) with lenvatinib, have shown enhanced therapeutic efficacy with improved objective response rates and survival outcomes compared to single-agent approaches [120].

These clinical achievements stress the critical role of inhibiting the VEGF pathway in HCC treatment and establish anti-angiogenic therapy as a cornerstone of modern HCC management.

Several key considerations should be taken into account when utilizing VEGF IHC in the diagnosis of HCC. Semiquantitative scoring systems (0–3 scale for staining intensity) might introduce interobserver variability, and there are currently no guidelines for evaluating VEGF expression in HCC. In addition, non-neoplastic cirrhotic hepatocytes frequently exhibit stronger VEGF expression (79.4%, 54/68 cases) than adjacent HCC (69.1%), complicating its diagnostic utility [116]. A solution to overcome these limitations would be the development of standardized IHC protocols, integration with molecular profiling, and correlation with clinical outcomes to optimize VEGF’s role in HCC management.

#### 3.2.2. CD31

CD31 (platelet endothelial cell adhesion molecule-1/PECAM-1) is a transmembrane glycoprotein expressed on endothelial cells, platelets, and leukocytes, mediating cell–cell adhesion and angiogenesis [121]. The primary role of CD31 IHC staining in HCC is to evaluate tumor angiogenesis, particularly through the quantification of microvascular density (MVD), which is essential for understanding tumor growth, progression, and metastatic potential. MVD is defined as the number of microvessels per unit area within tumor “hotspots.” Higher MVD, as determined by CD31 staining, is often associated with more aggressive tumor behavior and a poorer prognosis in HCC patients. A 2013 tissue microarray study of 135 HCC cases linked higher CD31 MVD to shorter RFS (26.5 vs. 56.6 months for high vs. low MVD) [122].

A 2022 study examined the clinicopathological features of HCC patients with the “vessels that encapsulate tumor clusters” (VETC) pattern, a distinctive vascular structure in HCC. By using CD31 staining to identify this pattern, researchers found that higher intra-tumoral MVD was associated with the VETC pattern, which independently predicted poorer long-term oncological outcomes. This underlines the prognostic significance of MVD in HCC, as tumors with higher MVD tend to be more aggressive and associated with worse survival outcomes [123].

## 4. Predictive Markers for Targeted Therapies

The increasing use of immune checkpoint inhibitors in HCC has highlighted the importance of immunohistochemical evaluation of PD-L1 and CTLA-4 as a crucial component in both prognostic assessment and therapeutic stratification. The current National Comprehensive Cancer Network guideline (NCCN version 1, 2025) recognizes the biological significance of PD-L1 and CTLA-4 pathways in HCC and includes immunotherapy agents targeting these checkpoints with atezolizumab (anti–PD-L1), durvalumab (anti–PD-L1), tremelimumab (anti–CTLA-4), and nivolumab plus ipilimumab (anti–PD-1/CTLA-4) as preferred or recommended options for advanced HCC [124]. However, there is no established role for routine PD-L1, CTLA-4, or other immune biomarker testing (including MSI, MMR, or TMB) in selecting patients for immunotherapy in HCC. Regulatory approvals for immune checkpoint inhibitors in HCC have been granted regardless of PD-L1 or CTLA-4 IHC status, as clinical trials have not consistently demonstrated predictive value for these markers in this context. The European Society for Medical Oncology (ESMO) supports the use of immune checkpoint inhibitors for advanced HCC as well, and notes the investigational nature of IHC biomarkers for therapy selection [125]. Ongoing studies are evaluating whether the quantitative or spatial analysis of PD-L1 and CTLA-4 by IHC could identify subgroups of HCC patients who are more likely to benefit from immunotherapy or to experience immune-related adverse events.

Table 3 summarizes currently used prognostic markers in HCC.

Predictive markers for targeted therapies in HCC have gained increasing attention with the emergence of new treatment options. The primary purpose of these markers is to identify patients who are more likely to respond to specific targeted therapies. This personalized approach helps optimize treatment strategies for maximal clinical benefits.

### 4.1. Programmed Cell Death Protein 1/Programmed Cell Death Ligand 1 (PD-1/PD-L1)

PD-1, a transmembrane receptor expressed on activated T cells, B cells, and myeloid cells, interacts with its ligand PD-L1—a surface glycoprotein upregulated on tumor cells and tumor-associated macrophages in HCC—to transmit inhibitory signals that suppress cytotoxic T-lymphocyte activity and promote immune tolerance [126].

In HCC, this interaction facilitates tumor immune escape by inducing T-cell exhaustion. The PD-1 protein expressed on activated T cells binds to PD-L1 expressed on HCC cells, Kupffer cells, and hepatocytes, transmitting inhibitory signals through immunoreceptor tyrosine-based switch motifs (ITSMs) to suppress cytotoxic T-cell activity [127,128]. This exhaustion is characterized by reduced cytokine production and impaired proliferative capacity, while simultaneously enhancing the survival of immunosuppressive regulatory T cells within the tumor microenvironment [129,130].

Anti-PD-1 and anti-PD-L1 antibodies are immunotherapy drugs that target the PD-1/PD-L1 pathway. By blocking the interaction between PD-1 and PD-L1, these drugs rescue immune cells to resume their anti-tumor activity, resulting in suppression of the tumor growth and improved patient survival [131,132].

A 2024 study examined the PD-L1 expression in advanced HCC. The researchers found that 25.4% of tumor tissues showed positive PD-L1 expression in tumor cells, while 53.7% exhibited PD-L1 positivity in immune cells. This study highlights the importance of assessing PD-L1 expression in both tumor and immune cell compartments for a more comprehensive evaluation [133].

A meta-analysis of 1843 HCC patients across 13 studies demonstrated PD-L1 positivity correlated with shorter overall survival (HR = 1.57) and poorer DFS (HR = 2.07), particularly in advanced Barcelona Clinical Liver Cancer (BCLC) stages [134]. This meta-analysis revealed critical associations between PD-L1 expression and key clinicopathological features that help explain its prognostic impact. High PD-L1 expression was significantly correlated with liver cirrhosis (OR = 1.66), indicating that PD-L1 upregulation may be linked to underlying liver dysfunction and chronic inflammatory processes. Most notably, elevated PD-L1 expression was associated with poorer BCLC staging (OR = 0.30), suggesting that PD-L1 upregulation becomes increasingly prominent in advanced disease stages where treatment options are limited and prognosis is poor. High PD-L1 expression also correlated with portal vein invasion (OR = 1.96), a feature associated with aggressive tumor behavior and metastatic potential.

However, research examining circulating PD-L1 levels in patients undergoing curative treatments revealed paradoxical associations, where higher circulating PD-L1 levels were linked to improved HCC-specific survival rather than worse outcomes [135]. This study of 81 HCC patients who underwent hepatic resection or liver transplantation found that elevated preoperative circulating PD-L1 (>700 pg/mL) was associated with improved survival (HR 0.12), directly contradicting the tissue-based findings. The authors proposed that in early-stage, resectable disease, high circulating PD-L1 may represent adaptive immune resistance against successful immune attack, whereas in advanced disease, elevated PD-L1 may reflect an overwhelming tumor burden and immune system failure.

Pembrolizumab monotherapy has received a category 2B recommendation based on the results of the KEYNOTE-224 (NCT02702414) and KEYNOTE-240 (NCT02702401) trials. KEYNOTE-224, a single-arm phase II study of 104 sorafenib-experienced patients, reported an ORR of 17% (3% complete responses) and a median OS of 13.2 months [132]. KEYNOTE-240, a phase III trial randomizing 413 patients to pembrolizumab or placebo, showed a numerical but non-significant OS improvement (13.9 vs. 10.6 months; HR 0.78, *p* = 0.0238) [131]. The efficacy of immune checkpoint therapy in HCC varies greatly among individuals, with only a small proportion of HCC patients responding positively. A major cause of immune resistance and poor efficacy in HCC patients is immune evasion, which is often due to insufficient infiltration of immune cells [136].However, the combination of anti-PD-L1 and anti-VEGF-A therapy has proved effective in HCC treatment (as mentioned above). The IMbrave150 trial (NCT03434379) established atezolizumab + bevacizumab as a category 1 preferred first-line regimen in the current NCCN Guidelines.

### 4.2. CTLA-4

CTLA-4 (cytotoxic T-lymphocyte-associated protein 4) is a transmembrane receptor expressed on activated CD4+ T cells and regulatory T cells (Tregs), functioning as a negative regulator of T-cell activation [137,138]. Mechanistically, CTLA-4 competes with CD28 for binding to B7 ligands (CD80/CD86) on antigen-presenting cells, thereby suppressing co-stimulatory signals required for T-cell activation [139].

In HCC, CTLA-4 overexpression is associated with immune evasion, mediated by Treg infiltration and suppression of effector T-cell responses [137]. CTLA-4 IHC staining in tumors localizes its expression on TILs (tumor-infiltrating lymphocytes) and, less frequently, on tumor cells [138].

CTLA-4 IHC serves as a prognostic and predictive tool. CTLA-4 IHC staining provides critical insights into HCC immunobiology, prognostic stratification, and therapeutic targeting, but varies by cellular compartment (TILs vs. tumor cells), which complicates prognostic interpretation [140]. Additionally, CTLA-4 staining helps identify patients who are likely to benefit from immune checkpoint inhibitors (ICIs).

A study of 112 HCC cases demonstrated that the quantity of CTLA-4+ on TILs correlated with high-grade tumors (Edmondson–Steiner grade III/IV), while CTLA-4 expression in tumor cells was linked to multiple lesions and lower tumor grades [140]. Stromal CTLA-4+ Treg infiltration has been linked to reduced CD8+ T-cell activity and poorer survival, highlighting the role of CTLA-4+ Treg in immune suppression [139,141].

The clinical evaluation of CTLA-4 inhibitors in HCC has progressed through several pivotal trials, revealing both the therapeutic potential and the challenges associated with this approach. One phase II trial (NCT01008358) investigated tremelimumab monotherapy in 20 patients with advanced HCV-related HCC, including individuals with Child–Pugh B cirrhosis (42.9%) and those refractory to sorafenib (23.8%). The participants achieved a partial response rate of 17.6% and a disease control rate of 76.4%, with a median OS of 8.2 months [142].

In the HIMALAYA phase III trial, 1171 unresectable HCC patients were randomized to receive either the STRIDE regimen (tremelimumab plus durvalumab), durvalumab monotherapy, or sorafenib. The STRIDE arm demonstrated superior median OS compared to that with sorafenib only (16.4 vs. 13.8 months; HR 0.78), with a 20.1% objective response rate versus 5.1% in the sorafenib group [143]. Four-year follow-up data further revealed a 25% long-term survival rate in STRIDE-treated patients, compared to that of 15.1% in the patients treated with sorafenib alone, spotlighting the durable response in a subset of individuals [143].

A separate study evaluating salvage anti-CTLA-4 therapy in 32 patients who progressed on prior anti-PD-1 agents reported a 22% objective response rate with nivolumab/ipilimumab combination therapy. However, this approach carried a significant toxicity burden, with 41% of patients experiencing immune-related adverse events (irAEs), including six cases of grade 3–4 events such as adrenal insufficiency and pneumonitis [144].

These trials collectively demonstrate that CTLA-4 inhibition, particularly in combination with PD-1/PD-L1 blockade, represents a meaningful therapeutic advance for HCC patients, with the STRIDE regimen now establishing a new standard of care for first-line treatment of unresectable disease. However, the substantial immune-related toxicity profile necessitates careful patient selection and close monitoring, particularly when considering salvage CTLA-4-based therapy in patients with compromised liver function or those who have failed prior checkpoint inhibitor treatment.

### 4.3. Microsatellite Instability-High (MSI-H)/Deficient Mismatch Repair (dMMR)

Microsatellite instability-high (MSI-H) and deficient mismatch repair (dMMR) status have emerged as critical predictive biomarkers in HCC, particularly in the context of immunotherapy. MSI-H arises from germline or somatic mutations in MMR genes (*EPCAM*, *MLH1*, *MSH2*, *MSH6*, and *PMS2*) or epigenetic silencing, leading to defective DNA repair and accumulation of frameshift mutations [145]. IHC for MMR proteins (MLH1, MSH2, MSH6, and PMS2) is the primary method to detect dMMR, with loss of nuclear staining indicating deficient protein expression.

In HCC, MSI-H occurs in approximately 2.0% of advanced cases, as demonstrated in a prospective cohort of 50 patients, where one case showed MSH2/MSH6 protein loss and PCR-confirmed MSI-H using BAT25 and NR24 markers [146]. Despite negative PD-L1 expression (<1%), this patient experienced tumor shrinkage with pembrolizumab, though another lesion showed resistance, suggesting intratumoral heterogeneity [146]. The MSI-H HCC cases [146] exhibited a high tumor mutation burden (TMB), CD8+ lymphocyte infiltration, and low VEGF expression, consistent with immunogenic microenvironments, as seen in other MSI-H cancers (such as colorectal cancer [147]).

The clinical application of MSI-H/dMMR testing in HCC faces several challenges. Most (97%) of HCC tumors are MMR proficient/MSI-stable, which limits the general utility of these markers as standalone indicators [148,149]. In a comprehensive analysis of 1306 HCC cases using next-generation sequencing and IHC, the overall prevalence of dMMR/MSI-H was found to be just 0.2%, with high TMB detected in only 5.1% of cases [150].

The inherent tumor heterogeneity characteristic of HCC further complicates MSI-H/dMMR applications, as different tumor clones may coexist within the same liver, potentially leading to false-negative results if MSI-H areas are not adequately represented in the biopsy specimen [146]. Technical aspects compound these issues, as discordances between IHC and PCR-based MSI testing can occur, necessitating careful correlation of results and potentially requiring both methodologies for accurate diagnosis [151].

Additionally, evidence gaps persist due to the lack of HCC-specific immunotherapy trials stratified by MSI status. HCC trials such as KEYNOTE-240 excluded MSI-H subanalyses, leaving efficacy estimates reliant on case reports. These limitations underscore the need for standardized IHC protocols, multimodal testing approaches, and dedicated trials.

**Table 3:** Prognostic markers in HCC.

Marker	Expression Pattern	Prognostic Significance	Therapeutic Implications	Limitations
p53 [105,106]	Nuclear accumulation in tumor cells Null pattern	Associates with aggressive tumor behavior; -Predicts early recurrence	Potential target for p53 pathway modulators (Amentoflavone). May enhance response to Donafenib.	Nonspecific staining in cirrhotic liver. Requires combination with molecular testing.
Ki-67 [41,109]	Nuclear staining in proliferating cells	LI > 10% predicts reduced 5-year survival. Correlates with vascular invasion.	Dual-energy CT shows promise for preoperative assessment. Guides adjuvant therapy selection.	Variable cutoff values (5–50%) cross studies. Affected by underlying liver pathology.
VEGF [113,116]	Cytoplasmic in tumor vessels	High expression: 48% 1-year recurrence. Serum levels > 500 pg/mL predict mVI.	Predicts response to bevacizumab/atezolizumab. Guides TACE–lenvatinib combinations.	Strong expression in cirrhotic hepatocytes. Semiquantitative scoring variability.
CD31 [110,122]	Endothelial cell membrane	Higher MVD correlates with shorter RFS (26.5 vs. 56.6 months). Associates with VETC pattern.	Potential biomarker for anti-angiogenic therapies. Helps identify vascular subtypes.	Lacks tumor specificity. Cross-reacts with platelets/macrophages.
PD-L1 [126,132,133]	Tumor cell membrane/immune cells	tumor cell+ (25.4%) correlates with reduced OS (HR = 1.57). immune cell+ (53.7%) correlates with resistance.	Guides pembrolizumab use. Combination with VEGF inhibitors shows synergy.	Spatial heterogeneity. Requires dual tumor/immune cell assessment.
CTLA-4 [139,144]	TILs and tumor cells	Stromal Treg infiltration correlates with poor CD8+ activity. Tumor cell expression correlates with multifocality.	STRIDE regimen shows durable responses. May benefit salvage nivolumab/ipilimumab.	Toxicity. Prognostic value varies by cellular localization.
MSI-H/dMMR [146,147,151]	Loss of MMR protein nuclear staining	2% prevalence in advanced HCC. High TMB despite PD-L1 negativity.	Pembrolizumab shows activity in case reports. Potential for neoantigen-directed therapies.	Low prevalence. Temporal and spatial heterogeneity.

## 5. Summary

While classical markers, such as HepPar1, Arg-1, and GPC3, remain foundational for distinguishing HCC from benign lesions and metastatic tumors, their limitations in specificity and sensitivity underscore the need for combinatorial approaches. For instance, Arg-1 demonstrates superior sensitivity (85.7%) in poorly differentiated HCC compared to HepPar1 (46.4%) [11], but its occasional reactivity in cholangiocarcinoma (6.9%) [152] necessitates integration with GPC3 (specificity 97.3%) or pCEA (canalicular pattern specificity). Albumin ISH, with 99% sensitivity for HCC detection, addresses gaps in high-grade tumors but shows cross-reactivity in 31.6% of ICCs, necessitating adjunct markers like CK19.

Recent advancements, such as the nine-factor IHC classifier (including PD-L1, CD8+, and macrophage markers), have further refined prognostic stratification in early-stage HCC, augmenting traditional systems like BCLC staging [153]. Additional prognostic indicators include cell cycle regulators such as Ki-67 and mutant p53. A Ki-67 labeling index > 10% predicts a 28.3% disparity in 5-year survival (54.3% vs. 82.6%), while p53 mutations correlate with epithelial–mesenchymal transition (EMT) and ferroptosis resistance. Angiogenesis-related markers, particularly VEGF, are also prognostically significant, with high VEGF expression (≥50% positive cells) correlating with decreased 5-year overall survival rates.

Predictive applications of prognostic markers have expanded with the advance of immunotherapy. PD-L1 expression in tumor cells and immune cells, as well as CTLA-4+ tumor-infiltrating lymphocytes, informs response to checkpoint inhibitors. Although MSI-H/dMMR status is rare in HCC (with a prevalence of 2%), it identifies candidates for exceptional immunotherapy responses despite technical challenges in detection. Recent research highlights the promising predictive value of novel biomarkers, such as long non-coding RNAs like ST8SIA6-AS1, Angiopoietin-like protein 4 (ANGPTL4), and Tyrosyl-tRNA synthetase 1 in HCC [154,155,156].

While immunohistochemistry has significantly advanced diagnostic precision in HCC, several critical limitations persist. Interobserver variability remains a significant obstacle, with substantial disagreement between pathologists even when using identical slides, particularly in small biopsy samples, where threshold assessments for immunoreactivity can produce discordant results [157,158]. Lack of standardization presents another fundamental challenge, as IHC suffers from variable consistency and poor reproducibility across laboratories due to under-fixation, irregular fixation protocols, minimal validation procedures, and the absence of standard interpretation systems [159]. These issues are compounded by limited availability in resource-constrained settings, where high maintenance costs, power fluctuations, expensive reagents, and supply chain delays create additional barriers to optimal implementation [160,161].

HCC sits at the crossroads of precision oncology and computational pathology. In recent years, artificial intelligence (AI) and machine learning have transformed immunohistochemistry (IHC) from a largely manual, semi-quantitative assay into a data-rich platform capable of yielding reproducible, multiplexed, and clinic-ready biomarkers [162]. Artificial intelligence (AI)-assisted IHC in HCC is poised to move beyond single-marker quantification toward a holistic, spatially resolved “digital immunogram” that integrates multiplexed chromogenic stains, RNA-ISH, and spatial transcriptomics under a unified analytical framework [163,164]. Advances in weakly supervised virtual staining will further democratize access by extracting surrogate biomarker information from routine H&E slides, obviating costly antibody panels and facilitating large-scale retrospective studies [165]. ML-enhanced IHC is reshaping the diagnostic, prognostic, and predictive landscape of hepatocellular carcinoma. By coupling quantitative accuracy with unprecedented scalability, AI promises to harmonize biomarker interpretation across pathologists and practice settings. Realizing this potential hinges on rigorous validation, transparent algorithms, and seamless integration into digital pathology ecosystems, initiatives are already underway but require sustained multidisciplinary collaboration. Future research directions hold promise for addressing these limitations. Multiplex IHC techniques enable simultaneous detection of multiple markers on single tissue sections, providing high-throughput, standardized quantitative analysis [166,167]. Integration with AI-based digital pathology represents a transformative opportunity, with artificial intelligence algorithms demonstrating remarkable accuracy in analyzing IHC-stained slides while reducing usage requirements and increasing diagnostic confidence [162].

IHC in HCC has transformed from a supplementary diagnostic tool to an integral component of precision oncology practice. The continued evolution of marker panels, technological platforms, and analytical approaches positions IHC as a cornerstone technology for advancing HCC care through improved diagnostic accuracy, refined prognostic assessment, and optimized therapeutic selection.

## Figures and Tables

**Table 1:** Currently used diagnostic markers for HCC.

Markers		Target/Antigen	Sensitivity (%)	Specificity (%)	Staining Pattern	Note
Hepatocellular lineage	HepPar1 [15,16,17,18,19]	CPS1 (mitochondrial)	70–87	71–97	Granular cytoplasmic	Stains non-hepatocellular tumors (e.g., gastrointestinal/hepatoid tumors)
	Arg-1 [15,20,21]	Urea cycle enzyme	76.6–96.0	97.5	Cytoplasmic	Rare negativity in well-differentiated HCC; occasional positivity in ICC and liver metastasis
	Albumin ISH [22,23,24]	Albumin mRNA	99	100 ‡	Cytoplasmic mRNA dots	Positivity in small-duct type ICC (31.6%) and hepatoid carcinomas
	CK8/18 [25,26]	Cytokeratins	54.5–83.3	Variable	Cytoplasmic and membrane	Hepatocellular cytokeratins but are not specific for hepatocellular lesions. Positive in most cancers and some sarcomas.
Liver architecture	CD10 [27,28]	Metalloproteinase	61–86	84–87	Canalicular/membranous	Expressed in focal nodular hyperplasia (FNH, 100%) and ICC (31.6%)
	pCEA [29,30]	Biliary glycoprotein	60–90	95–100 *	Canalicular	Declining sensitivity in poorly diff HCC; technical variability
Malignant hepatocyte	GPC3 [31,32,33,34,35]	Cell-surface proteoglycan	63–92	94–100	Cytoplasmic/membranous (heterogeneous)	Reduced sensitivity in well-differentiated HCC; positivity in ICC (14–21%)
	GS [36,37,38]	β-catenin pathway	43.9–100	90–94.3	Diffuse cytoplasmic	Increased expression in cirrhosis and chronic hepatitis B virus-infected liver; focal positivity in dysplastic nodules
	HSP70 [37,39,40]	Stress response protein	57.5–78.2	85–100	Nuclear/cytoplasmic	Positivity in liver metastatic adenocarcinoma and ICC; heterogeneity in poorly differentiated HCCs
	AFP [34,35]	Oncoprotein	30% (2–62%)	90%	Cytoplasmic	Positive in yolk sac tumor, hepatoblastoma, hepatoid tumor, gastric carcinoma, pancreatic adenocarcinoma, infantile hemangioendothelioma, cirrhosis, chronic hepatitis B, and other liver diseases.
Others	Ki-67 [41]	Cell proliferation	N/A	N/A	Nuclear	Higher in malignant cells than in background hepatocytes
	CD34 [42,43]	Sinusoidal endothelium	92.8	85–90	Diffuse sinusoidal capillarization	Patchy staining in well-differentiated HCC and cirrhosis
	CK19 [44,45]	Biliary/progenitor cells	15	95.6 †	Cytoplasmic and membrane	Indicates aggressive subtypes; not HCC-specific

* Specificity for canalicular pattern only. † When combined with AFP. ‡ Specificity for hepatic origin vs. non-hepatic metastases. GS, glutamine synthetase. HSP70, heat shock protein 70.

**Table 2:** Performance of IHC markers of HCC at GML [94].

Markers or Antibodies	GML Data %(N)
Hep Par1	94% (18)
Arginase-1	98% (17/18)
Glypican-3	72% (13/18)
pCEA	94% (17/18)
CD10	61% (11/18)
CK19	6% (1/18)
CD34	100% (18/18)

## Data Availability

The original contributions presented in this study are included in the article. Further inquiries can be directed to the corresponding authors.

## References

[1] Bray F., Ferlay J., Soerjomataram I., Siegel R.L., Torre L.A., Jemal A. (2018). Global cancer statistics 2018: GLOBOCAN estimates of incidence and mortality worldwide for 36 cancers in 185 countries. CA Cancer J. Clin..

[2] Llovet J.M., Kelley R.K., Villanueva A., Singal A.G., Pikarsky E., Roayaie S., Lencioni R., Koike K., Zucman-Rossi J., Finn R.S. (2021). Hepatocellular carcinoma. Nat. Rev. Dis. Primers.

[3] Choi J.H., Thung S.N. (2023). Advances in Histological and Molecular Classification of Hepatocellular Carcinoma. Biomedicines.

[4] Toh M.R., Wong E.Y.T., Wong S.H., Ng A.W.T., Loo L.-H., Chow P.K.-H., Ngeow J. (2023). Global Epidemiology and Genetics of Hepatocellular Carcinoma. Gastroenterology.

[5] Eslam M., Newsome P.N., Sarin S.K., Anstee Q.M., Targher G., Romero-Gomez M., Zelber-Sagi S., Wai-Sun Wong V., Dufour J.-F., Schattenberg J.M. (2020). A new definition for metabolic dysfunction-associated fatty liver disease: An international expert consensus statement. J. Hepatol..

[6] Motta B.M., Masarone M., Torre P., Persico M. (2023). From Non-Alcoholic Steatohepatitis (NASH) to Hepatocellular Carcinoma (HCC): Epidemiology, Incidence, Predictions, Risk Factors, and Prevention. Cancers.

[7] Kale S.R., Karande G., Gudur A., Garud A., Patil M.S., Patil S. (2024). Recent Trends in Liver Cancer: Epidemiology, Risk Factors, and Diagnostic Techniques. Cureus.

[8] Bittaye S.O., Njie R., Tekanyi M., Kambi A., Tamba S., Fatty G., Bah Y., Jatta A., Sanneh L., Sisawo M.M. (2020). Abstract 2346: Clinical manifestation, staging and prognosis of hepatocellular carcinoma in Gambian patients. Cancer Res..

[9] Prasad D., Nguyen M.H. (2021). Epidemiology, pathogenesis, diagnosis, surveillance, and management of hepatocellular carcinoma associated with vascular liver disease. Kaohsiung J. Med. Sci..

[10] Wazir H., Abid M., Essani B., Saeed H., Ahmad Khan M., Nasrullah F., Qadeer U., Khalid A., Varrassi G., Muzammil M.A. (2023). Diagnosis and Treatment of Liver Disease: Current Trends and Future Directions. Cureus.

[11] Mocan L.P., Rusu I., Melincovici C.S., Boșca B.A., Mocan T., Crăciun R., Spârchez Z., Iacobescu M., Mihu C.M. (2023). The Role of Immunohistochemistry in the Differential Diagnosis between Intrahepatic Cholangiocarcinoma, Hepatocellular Carcinoma and Liver Metastasis, as Well as Its Prognostic Value. Diagnostics.

[12] Kmeid M., Park Y.N., Chung T., Pacheco R.R., Arslan M.E., Lee H. (2023). SEPT9 Expression in Hepatic Nodules: An Immunohistochemical Study of Hepatocellular Neoplasm and Metastasis. Appl. Immunohistochem. Mol. Morphol..

[13] Xu C., Zhou G., Zheng B., Lu G., Shao X., Tao C., Xu X., Wang J., Pan C., Zhang Z. (2016). Decreased expression of iron regulatory protein-1 in hepatocellular carcinoma associates with poor prognosis. Int. J. Clin. Exp. Pathol..

[14] Dai Z., Wang Y., Sun N., Zhang C. (2024). Characterizing ligand-receptor interactions and unveiling the pro-tumorigenic role of CCL16-CCR1 axis in the microenvironment of hepatocellular carcinoma. Front. Immunol..

[15] Yan B.C., Gong C., Song J., Krausz T., Tretiakova M., Hyjek E., Al-Ahmadie H., Alves V., Xiao S.-Y., Anders R.A. (2010). Arginase-1: A new immunohistochemical marker of hepatocytes and hepatocellular neoplasms. Am. J. Surg. Pathol..

[16] Radwan N.A., Ahmed N.S. (2012). The diagnostic value of arginase-1 immunostaining in differentiating hepatocellular carcinoma from metastatic carcinoma and cholangiocarcinoma as compared to HepPar-1. Diagn. Pathol..

[17] Fujiwara M., Kwok S., Yano H., Pai R.K. (2012). Arginase-1 is a more sensitive marker of hepatic differentiation than HepPar-1 and glypican-3 in fine-needle aspiration biopsies. Cancer Cytopathol..

[18] McKnight R., Nassar A., Cohen C., Siddiqui M.T. (2012). Arginase-1: A novel immunohistochemical marker of hepatocellular differentiation in fine needle aspiration cytology. Cancer Cytopathol..

[19] Larson B.K., Dhall D., Guindi M. (2024). Arginase-1 is More Specific Than Hepatocyte Paraffin 1 for Differentiating Hepatocellular Carcinomas with Cytoplasmic Clearing from Nonhepatocellular Clear Cell Tumors in Liver Biopsies. Appl. Immunohistochem. Mol. Morphol..

[20] Wang C., Shao X., Zhang X., Xie C., Yu J., Xu X., Yang J., Li Y., Xu W. (2020). Diagnostic value of glypican-3, arginase-1 and hepatocyte paraffin antigen-1 in differentiating hepatocellular carcinoma from intrahepatic cholangiocarcinoma. Transl. Cancer Res..

[21] Obiorah I.E., Chahine J., Park B.U., Ko K., de Guzman J., Kallakury B. (2019). Well differentiated arginase-1 negative hepatocellular carcinoma. Transl. Gastroenterol. Hepatol..

[22] Shahid M., Mubeen A., Tse J., Kakar S., Bateman A., Borger D., Rivera M., Ting D.T., Deshpande V. (2015). Branched chain in situ hybridization for albumin as a marker of hepatocellular differentiation: Evaluation of manual and automated in situ hybridization platforms. Am. J. Surg. Pathol..

[23] Ferrone C.R., Ting D.T., Shahid M., Konstantinidis I.T., Sabbatino F., Goyal L., Rice-Stitt T., Mubeen A., Arora K., Bardeesey N. (2016). The Ability to Diagnose Intrahepatic Cholangiocarcinoma Definitively Using Novel Branched DNA-Enhanced Albumin RNA In Situ Hybridization Technology. Ann. Surg. Oncol..

[24] Nasir A., Lehrke H.D., Mounajjed T., Said S., Zhang L., Yasir S., Shah S.S., Chandan V.S., Smyrk T.C., Moreira R.K. (2019). Albumin In Situ Hybridization Can Be Positive in Adenocarcinomas and Other Tumors From Diverse Sites. Am. J. Clin. Pathol..

[25] Yan J., Yang A., Tu S. (2024). The relationship between keratin 18 and epithelial-derived tumors: As a diagnostic marker, prognostic marker, and its role in tumorigenesis. Front. Oncol..

[26] Ceausu M., Socea B., Serban D., Smarandache C.G., Predescu D., Bacalbaşa N., Slavu I., Tulin A., Alecu L., Ceauşu Z. (2021). Heterogeneity of antigenic constellation in human hepatocellular carcinoma. Exp. Ther. Med..

[27] Lin F., Abdallah H., Meschter S. (2004). Diagnostic utility of CD10 in differentiating hepatocellular carcinoma from metastatic carcinoma in fine-needle aspiration biopsy (FNAB) of the liver. Diagn. Cytopathol..

[28] Wen C., Huang C., Chen S., Liu X., Yin W., Tao L. (2025). Membranous Staining of CD10 Is Related to Steatosis Changes in Hepatocellular Carcinoma: An Investigation of CD10 Stainning in Hepatocellular Carcinoma, Focal Nodular Hyperplasia, and Intrahepatic Cholangiocarcinoma. Appl. Immunohistochem. Mol. Morphol..

[29] Wang L., Vuolo M., Suhrland M.J., Schlesinger K. (2006). HepPar1, MOC-31, pCEA, mCEA and CD10 for distinguishing hepatocellular carcinoma vs. metastatic adenocarcinoma in liver fine needle aspirates. Acta Cytol..

[30] El-Rebey H., Kandil M., El-Azab D., Abd-Elhamed S., Hemida A. (2019). Bile salt export pump, arginase 1, and hepatocyte paraffin 1 expression in differential diagnosis of hepatocellular carcinoma from nonhepatocellular carcinoma. Egypt. J. Pathol..

[31] Shafizadeh N., Ferrell L.D., Kakar S. (2008). Utility and limitations of glypican-3 expression for the diagnosis of hepatocellular carcinoma at both ends of the differentiation spectrum. Mod. Pathol..

[32] Kaseb A.O., Hassan M., Lacin S., Abdel-Wahab R., Amin H.M., Shalaby A., Wolff R.A., Yao J., Rashid A., Vennapusa B. (2016). Evaluating clinical and prognostic implications of Glypican-3 in hepatocellular carcinoma. Oncotarget.

[33] Guo M., Zhang H., Zheng J., Liu Y. (2020). Glypican-3: A New Target for Diagnosis and Treatment of Hepatocellular Carcinoma. J. Cancer.

[34] Lau S.K., Prakash S., Geller S.A., Alsabeh R. (2002). Comparative immunohistochemical profile of hepatocellular carcinoma, cholangiocarcinoma, and metastatic adenocarcinoma. Hum. Pathol..

[35] Ulbright T.M. (2018). Pitfalls in the interpretation of specimens from patients with testicular tumours, with an emphasis on variant morphologies. Pathology.

[36] Ye Y., Yu B., Wang H., Yi F. (2023). Glutamine metabolic reprogramming in hepatocellular carcinoma. Front. Mol. Biosci..

[37] Lagana S.M., Salomao M., Bao F., Moreira R.K., Lefkowitch J.H., Remotti H.E. (2013). Utility of an immunohistochemical panel consisting of glypican-3, heat-shock protein-70, and glutamine synthetase in the distinction of low-grade hepatocellular carcinoma from hepatocellular adenoma. Appl. Immunohistochem. Mol. Morphol..

[38] Shao M., Tao Q., Xu Y., Xu Q., Shu Y., Chen Y., Shen J., Zhou Y., Wu Z., Chen M. (2023). Glutamine synthetase-negative hepatocellular carcinoma has better prognosis and response to sorafenib treatment after hepatectomy. Chin. Med. J..

[39] Tremosini S., Forner A., Boix L., Vilana R., Bianchi L., Reig M., Rimola J., Rodríguez-Lope C., Ayuso C., Solé M. (2012). Prospective validation of an immunohistochemical panel (glypican 3, heat shock protein 70 and glutamine synthetase) in liver biopsies for diagnosis of very early hepatocellular carcinoma. Gut.

[40] Joo M., Chi J.G., Lee H. (2005). Expressions of HSP70 and HSP27 in Hepatocellular Carcinoma. J. Korean Med. Sci..

[41] King K.L., Hwang J.J., Chau G.Y., Tsay S.H., Chi C.W., Lee T.G., Wu L.H., Wu C.W., Lui W.Y. (1998). Ki-67 expression as a prognostic marker in patients with hepatocellular carcinoma. J. Gastroenterol. Hepatol..

[42] Yao S., Zhang J., Chen H., Sheng Y., Zhang X., Liu Z., Zhang C. (2013). Diagnostic value of immunohistochemical staining of GP73, GPC3, DCP, CD34, CD31, and reticulin staining in hepatocellular carcinoma. J. Histochem. Cytochem..

[43] Cui S., Hano H., Sakata A., Harada T., Liu T., Takai S., Ushigome S. (1996). Enhanced CD34 expression of sinusoid-like vascular endothelial cells in hepatocellular carcinoma. Pathol. Int..

[44] Cai X., Feng L., Liu H., Xu M., Qu Y., Wan X., Gao C., Lu L. (2016). Cytokeratin19 positive hepatocellular carcinoma is associated with increased peritumoral ductular reaction. Ann. Hepatol..

[45] Chen Y., Chen J., Zhang Y., Lin Z., Wang M., Huang L., Huang M., Tang M., Zhou X., Peng Z. (2021). Preoperative Prediction of Cytokeratin 19 Expression for Hepatocellular Carcinoma with Deep Learning Radiomics Based on Gadoxetic Acid-Enhanced Magnetic Resonance Imaging. J. Hepatocell. Carcinoma.

[46] Villari D., Caruso R., Grosso M., Vitarelli E., Righi M., Barresi G. (2002). Hep Par 1 in gastric and bowel carcinomas: An immunohistochemical study. Pathology.

[47] Mattiolo P., Scarpa A., Luchini C. (2023). Hepatoid tumors of the gastrointestinal/pancreatobiliary district: Morphology, immunohistochemistry, and molecular profiles. Hum. Pathol..

[48] Thamboo T.P., Wee A. (2004). Hep Par 1 expression in carcinoma of the cervix: Implications for diagnosis and prognosis. J. Clin. Pathol..

[49] Bao S., Gu J., Gan K., Fang Y., Wang T., Lin J., Zeng Z., Huang H. (2021). Glypican-3 and hepatocyte paraffin-1 combined with alpha-fetoprotein as a novel risk scoring model for predicting early recurrence of hepatocellular carcinoma after curative resection. Eur. J. Gastroenterol. Hepatol..

[50] Yussif S.M., Elzeftawy D.H., Elshawaf I.M., Elkashef W.F. (2022). Role of arginase 1 immunohistochemical marker in differentiating hepatocellular carcinoma from other primary and secondary carcinomas of the liver, a tissue microarray study. Indian J. Pathol. Oncol..

[51] Bin-shen W. (2014). Effects of arginase-1 in identifying primary hepatocellular carcinoma and hepatic metastasis. Carcinog. Mutagen..

[52] Chandan V.S., Shah S.S., Torbenson M.S., Wu T.-T. (2016). Arginase-1 is frequently positive in hepatoid adenocarcinomas. Hum. Pathol..

[53] Xiao W.-K., Qi C.-Y., Chen D., Li S.-Q., Fu S.-J., Peng B.-G., Liang L.-J. (2014). Prognostic significance of glypican-3 in hepatocellular carcinoma: A meta-analysis. BMC Cancer.

[54] Moudi B., Heidari Z., Mahmoudzadeh-Sagheb H. (2019). Meta-analysis and systematic review of prognostic significance of Glypican-3 in patients with hepatitis B-related hepatocellular carcinoma. Virusdisease.

[55] Chen I.-P., Ariizumi S., Nakano M., Yamamoto M. (2014). Positive glypican-3 expression in early hepatocellular carcinoma predicts recurrence after hepatectomy. J. Gastroenterol..

[56] Zheng X., Liu X., Lei Y., Wang G., Liu M. (2022). Glypican-3: A Novel and Promising Target for the Treatment of Hepatocellular Carcinoma. Front. Oncol..

[57] Cheng A.-L., Yen C.-J., Okusaka T., Ikeda M., Hsu C.-H., Wu S.-Y., Morizane C., Hashimoto Y., Ueshima K., Ohtomo T. (2018). A phase I, open-label, multi-center, dose-escalation study of codrituzumab, an anti-glypican-3 monoclonal antibody, in combination with atezolizumab in patients with locally advanced or metastatic hepatocellular carcinoma. Ann. Oncol..

[58] Ho M., Kim H. (2011). Glypican-3: A new target for cancer immunotherapy. Eur. J. Cancer Oxf. Engl..

[59] Baumhoer D., Tornillo L., Stadlmann S., Roncalli M., Diamantis E.K., Terracciano L.M. (2008). Glypican 3 expression in human nonneoplastic, preneoplastic, and neoplastic tissues: A tissue microarray analysis of 4387 tissue samples. Am. J. Clin. Pathol..

[60] Ibrahim T.R., Abdel-Raouf S.M. (2015). Immunohistochemical Study of Glypican-3 and HepPar-1 in Differentiating Hepatocellular Carcinoma from Metastatic Carcinomas in FNA of the Liver. Pathol. Oncol. Res..

[61] Man X.-B., Tang L., Zhang B.-H., Li S.-J., Qiu X.-H., Wu M.-C., Wang H.-Y. (2005). Upregulation of Glypican-3 expression in hepatocellular carcinoma but downregulation in cholangiocarcinoma indicates its differential diagnosis value in primary liver cancers. Liver Int..

[62] Geramizadeh B., Seirfar N. (2015). Diagnostic Value of Arginase-1 and Glypican-3 in Differential Diagnosis of Hepatocellular Carcinoma, Cholangiocarcinoma and Metastatic Carcinoma of Liver. Hepat. Mon..

[63] Li J., Wei L. (2014). Significance of arginase-1, glypican-3, hepatocyte paraffin antigen 1 and alpha-fetoprotein in diagnosis and differential diagnosis of liver tumors. Chin. J. Pathol..

[64] Wee A. (2006). Diagnostic utility of immunohistochemistry in hepatocellular carcinoma, its variants and their mimics. Appl. Immunohistochem. Mol. Morphol..

[65] Kakar S., Gown A.M., Goodman Z.D., Ferrell L.D. (2007). Best Practices in Diagnostic Immunohistochemistry: Hepatocellular Carcinoma Versus Metastatic Neoplasms. Arch. Pathol. Lab. Med..

[66] Takahashi Y., Dungubat E., Kusano H., Ganbat D., Tomita Y., Odgerel S., Fukusato T. (2021). Application of Immunohistochemistry in the Pathological Diagnosis of Liver Tumors. Int. J. Mol. Sci..

[67] Morrison C., Marsh W., Frankel W.L. (2002). A Comparison of CD10 to pCEA, MOC-31, and Hepatocyte for the Distinction of Malignant Tumors in the Liver. Mod. Pathol..

[68] Nguyen T., Phillips D., Jain D., Torbenson M., Wu T.-T., Yeh M.M., Kakar S. (2015). Comparison of 5 Immunohistochemical Markers of Hepatocellular Differentiation for the Diagnosis of Hepatocellular Carcinoma. Arch. Pathol. Lab. Med..

[69] Dilek O.N., Kahraman D.İ.A., Kahraman G. (2024). Carcinoembryonic antigen in the diagnosis, treatment, and follow-up of focal liver lesions. World J. Gastrointest. Surg..

[70] Wang S., Xiao Y., An X., Luo L., Gong K., Yu D. (2024). A comprehensive review of the literature on CD10: Its function, clinical application, and prospects. Front. Pharmacol..

[71] Ahuja A., Gupta N., Kalra N., Srinivasan R., Chawla Y., Rajwanshi A. (2008). Role of CD10 immunochemistry in differentiating hepatocellular carcinoma from metastatic carcinoma of the liver. Cytopathology.

[72] Kakehashi A., Inoue M., Wei M., Fukushima S., Wanibuchi H. (2009). Cytokeratin 8/18 overexpression and complex formation as an indicator of GST-P positive foci transformation into hepatocellular carcinomas. Toxicol. Appl. Pharmacol..

[73] Ismail S.A., Saadany S.E., Ziada D.H., Zakaria S.S., Mayah W.W., Elashry H., Arafa M., Elmashad N. (2017). Cytokeratin-18 in Diagnosis of HCC in Patients with Liver Cirrhosis. Asian Pac. J. Cancer Prev..

[74] Eguchi A., Iwasa M., Tamai Y., Yamada M., Okuno K., Shigefuku R., Yoshikawa K., Tempaku M., Sakaguchi K., Tanaka H. (2022). The prognostic potential of fragmented CK18 serum levels in HCC patients reflecting disease progression and overall hepatocyte damage. Front. Oncol..

[75] Su H., Han C., He Y., Liang T., Mo S., Yang C., Liao X., Zhu G., Ye X., Peng T. (2021). Molecular mechanism of CK19 involved in the regulation of postoperative recurrence of HBV-associated primary hepatocellular carcinoma in Guangxi. Ann. Transl. Med..

[76] Zhuo J., Lu D., Lin Z., Yang X., Yang M., Wang J., Tao Y., Wen X., Li H., Lian Z. (2021). The distinct responsiveness of cytokeratin 19-positive hepatocellular carcinoma to regorafenib. Cell Death Dis..

[77] Zhao Y., Tan X., Chen J., Tan H., Huang H., Luo P., Liang Y., Jiang X. (2023). Preoperative prediction of cytokeratin-19 expression for hepatocellular carcinoma using T1 mapping on gadoxetic acid-enhanced MRI combined with diffusion-weighted imaging and clinical indicators. Front. Oncol..

[78] Di Tommaso L., Roncalli M. (2017). Tissue Biomarkers in Hepatocellular Tumors: Which, When, and How. Front. Med..

[79] Long J., Wang H., Lang Z., Wang T., Long M., Wang B. (2010). Expression level of glutamine synthetase is increased in hepatocellular carcinoma and liver tissue with cirrhosis and chronic hepatitis B. Hepatol. Int..

[80] Moudi B., Heidari Z., Mahmoudzadeh-Sagheb H., Alavian S.-M., Lankarani K.B., Farrokh P., Nyengaard J.R. (2018). Concomitant use of heat-shock protein 70, glutamine synthetase and glypican-3 is useful in diagnosis of HBV-related hepatocellular carcinoma with higher specificity and sensitivity. Eur. J. Histochem..

[81] Di Tommaso L., Destro A., Seok J.Y., Balladore E., Terracciano L., Sangiovanni A., Iavarone M., Colombo M., Jang J.J., Yu E. (2009). The application of markers (HSP70 GPC3 and GS) in liver biopsies is useful for detection of hepatocellular carcinoma. J. Hepatol..

[82] Wang C., Zhang Y., Guo K., Wang N., Jin H., Liu Y., Qin W. (2016). Heat shock proteins in hepatocellular carcinoma: Molecular mechanism and therapeutic potential. Int. J. Cancer.

[83] Cho W., Jin X., Pang J., Wang Y., Mivechi N.F., Moskophidis D. (2019). The Molecular Chaperone Heat Shock Protein 70 Controls Liver Cancer Initiation and Progression by Regulating Adaptive DNA Damage and Mitogen-Activated Protein Kinase/Extracellular Signal-Regulated Kinase Signaling Pathways. Mol. Cell. Biol..

[84] Paul R., Shreya S., Pandey S., Shriya S., Abou Hammoud A., Grosset C.F., Prakash Jain B. (2024). Functions and Therapeutic Use of Heat Shock Proteins in Hepatocellular Carcinoma. Livers.

[85] Mohamed S.A., Tealeb A.-S.M.I. (2022). The role of heat shock protein 70 and glypican 3 expression in early diagnosis of hepatocellular carcinoma. Egypt. J. Pathol..

[86] Nguyen T.B., Roncalli M., Di Tommaso L., Kakar S. (2016). Combined use of heat-shock protein 70 and glutamine synthetase is useful in the distinction of typical hepatocellular adenoma from atypical hepatocellular neoplasms and well-differentiated hepatocellular carcinoma. Mod. Pathol..

[87] Di Tommaso L., Franchi G., Park Y.N., Fiamengo B., Destro A., Morenghi E., Montorsi M., Torzilli G., Tommasini M., Terracciano L. (2007). Diagnostic value of HSP70, glypican 3, and glutamine synthetase in hepatocellular nodules in cirrhosis. Hepatology.

[88] Choi J.H., Thung S.N. (2024). Pathology and diagnostic approaches to well-differentiated hepatocellular lesions: A narrative review. J. Yeungnam Med. Sci..

[89] Zhou C., Zhang X., Zhou K., Hou Y., Chen F., Zhang X., Ji Y., Qiu S., Fan J., Zhou J. (2022). Long-term outcomes and prognosis for patients with sarcomatoid hepatocellular carcinoma. Ann. Transl. Med..

[90] Chen X., Li S., Jiang Z.-M., Gu M., Feng B., Xie Z.-Q., Huang M.-Y., Chen Z., Tang M.-L. (2023). C-type Lectin Domain Family 4 Member G (CLEC4G) Is a Negative Marker for CD34 in the Evolution of Liver Pathogenesis. Ann. Clin. Lab. Sci..

[91] Di Carlo I., Fraggetta F., Lombardo R., Azzarello G., Vasquez E., Puleo S. (2002). CD 34 expression in chronic and neoplastic liver diseases. Panminerva Med..

[92] Kalyani P., Sarma A., Gupta S., Ahmed S., Kakoti L. (2024). Role of CD34, SMA & Ki-67 Immunohistochemistry– An Important Diagnostic Tool in Differentiating Early Well Differentiated Hepatocellular Carcinoma from Benign Hepatic Mimickers: A Retrospective Study from North East India. Sch. J. Appl. Med. Sci..

[93] Fu X., Yang Y., Zhang D. (2022). Molecular mechanism of albumin in suppressing invasion and metastasis of hepatocellular carcinoma. Liver Int..

[94] (2022). Handbook of Practical Immunohistochemistry: Frequently Asked Questions.

[95] Krings G., Ramachandran R., Jain D., Wu T.-T., Yeh M.M., Torbenson M., Kakar S. (2013). Immunohistochemical pitfalls and the importance of glypican 3 and arginase in the diagnosis of scirrhous hepatocellular carcinoma. Mod. Pathol..

[96] Ward S.C., Huang J., Tickoo S.K., Thung S.N., Ladanyi M., Klimstra D.S. (2010). Fibrolamellar carcinoma of the liver exhibits immunohistochemical evidence of both hepatocyte and bile duct differentiation. Mod. Pathol..

[97] Murakata L.A., Ishak K.G., Nzeako U.C. (2000). Clear Cell Carcinoma of the Liver: A Comparative Immunohistochemical Study with Renal Clear Cell Carcinoma. Mod. Pathol..

[98] Numbere N., Zhang D., Agostini-Vulaj D. (2021). A rare histologic subtype of hepatocellular carcinoma, sarcomatoid hepatocellular carcinoma: Report of a case. Hepatic Oncol..

[99] Luo C., Xin H., Yin D., Zhao T., Hu Z., Zhou Z., Sun R., Yao N., Sun Q., Fan J. (2021). Characterization of immune infiltration in sarcomatoid hepatocellular carcinoma. Aging.

[100] Paslaru L., Bindea G., Nastase A., Sorop A., Zimbru C., Herlea V., Hrehoret D., Brasoveanu V., Zamfir R., Dima S. (2022). Comparative RNA-Sequencing Analysis Reveals High Complexity and Heterogeneity of Transcriptomic and Immune Profiles in Hepatocellular Carcinoma Tumors of Viral (HBV, HCV) and Non-Viral Etiology. Medicina.

[101] Jean K., Tawheed A., Nguyen L.B.L., Heikal T., Eldaly U., Elhadidy N.G., Elghaieb A., Aboudonia A., Tondeur L., Dublineau A. (2024). A Comparison of Presentation, Treatment, and Survival After Hepatocellular Carcinoma of Viral and Non-Viral Etiology in Damietta, Egypt, 2007–2019. J. Hepatocell. Carcinoma.

[102] Cai M., Hurwitz K., Hendrickson K., Jackman L., Yu Y., Ng J., Henkel A., Ferlini C. (2025). Incidence of viral and non-viral etiologies of hepatocellular carcinoma (HCC) in the US over time by race and ethnicity. J. Clin. Oncol..

[103] Jernigan P.L., Wima K., Hanseman D.J., Hoehn R.S., Ahmad S.A., Shah S.A., Abbott D.E. (2015). Natural history and treatment trends in hepatocellular carcinoma subtypes: Insights from a national cancer registry. J. Surg. Oncol..

[104] Gomes A.S., Ramos H., Inga A., Sousa E., Saraiva L. (2021). Structural and Drug Targeting Insights on Mutant p53. Cancers.

[105] Xu J., Yan B., Xiao X., Yuan Q., Dong X., Du Q., Zhang J., Shan L., Ding Z., Zhou L. (2021). Green Tea-Derived Theabrownin Induces Cellular Senescence and Apoptosis of Hepatocellular Carcinoma via P53 Signaling-Mediated Mechanism Bypassed by JNK Regulation. Res. Sq..

[106] Rahadiani N., Stephanie M., Perkasa A.G., Handjari D.R., Krisnuhoni E. (2023). p53 expression is associated with tumor stage, grade and subtype in patients with hepatocellular carcinoma. Mol. Clin. Oncol..

[107] Wang X., Li L., Wang L., Chen M. (2023). The expression of Ki-67 and Glypican-3 in hepatocellular carcinoma was evaluated by comparing DWI and 18F-FDG PET/CT. Front. Oncol..

[108] Luo Y., Ren F., Liu Y., Shi Z., Tan Z., Xiong H., Dang Y., Chen G. (2015). Clinicopathological and prognostic significance of high Ki-67 labeling index in hepatocellular carcinoma patients: A meta-analysis. Int. J. Clin. Exp. Med..

[109] Karabulut E., Akbulut S., Samdanci E.T., Akatli A.N., Elsarawy A., Kucukakcali Z., Ogut Z., Tuncer A., Ince V., Yilmaz S. (2025). Are Ki-67 and Procalcitonin Expression Levels Useful in Predicting the Biological Behavior of Hepatocellular Carcinoma After Liver Transplantation?. J. Clin. Med..

[110] Ramos-Santillan V., Oshi M., Nelson E., Endo I., Takabe K. (2024). High Ki67 Gene Expression Is Associated with Aggressive Phenotype in Hepatocellular Carcinoma. World J. Oncol..

[111] Lei H.-J., Wang S.-Y., Chau I.Y., Li A.F.-Y., Chau Y.-P., Hsia C.-Y., Chou S.-C., Kao Y.-C., Chau G.-Y. (2021). Hepatoma upregulated protein and Ki-67 expression in resectable hepatocellular carcinoma. J. Chin. Med. Assoc..

[112] Yasir S., Chen Z.E., Said S., Wu T.-T., Torbenson M. (2021). Biopsies of Hepatocellular Carcinoma with no Reticulin Loss: An Important Diagnostic Pitfall. Hum. Pathol..

[113] Poon R.T.-P., Ng I.O.-L., Lau C., Zhu L.-X., Yu W.-C., Lo C.-M., Fan S.-T., Wong J. (2001). Serum Vascular Endothelial Growth Factor Predicts Venous Invasion in Hepatocellular Carcinoma : A Prospective Study. Ann. Surg..

[114] Pocino K., Napodano C., Marino M., Di Santo R., Miele L., De Matthaeis N., Gulli F., Saporito R., Rapaccini G.L., Ciasca G. (2021). A Comparative Study of Serum Angiogenic Biomarkers in Cirrhosis and Hepatocellular Carcinoma. Cancers.

[115] von Marschall Z., Cramer T., Höcker M., Finkenzeller G., Wiedenmann B., Rosewicz S. (2001). Dual mechanism of vascular endothelial growth factor upregulation by hypoxia in human hepatocellular carcinoma. Gut.

[116] Deli G., Jin C.-H., Mu R., Yang S., Liang Y., Chen D., Makuuchi M. (2005). Immunohistochemical assessment of angiogenesis in hepatocellular carcinoma and surrounding cirrhotic liver tissues. World J. Gastroenterol..

[117] Choi S.B., Han H.J., Kim W.B., Song T.J., Choi S.Y. (2017). VEGF overexpression predicts poor survival in hepatocellular carcinoma. Open Med..

[118] Cheng A.L., Qin S., Ikeda M., Galle P.R., Ducreux M., Kim T.Y., Lim H.Y., Kudo M., Breder V., Merle P. (2022). Updated efficacy and safety data from IMbrave150: Atezolizumab plus bevacizumab vs. sorafenib for unresectable hepatocellular carcinoma. J. Hepatol..

[119] Zhu A.X., Kang Y.K., Yen C.J., Finn R.S., Galle P.R., Llovet J.M., Assenat E., Brandi G., Pracht M., Lim H.Y. (2019). Ramucirumab after sorafenib in patients with advanced hepatocellular carcinoma and increased α-fetoprotein concentrations (REACH-2): A randomised, double-blind, placebo-controlled, phase 3 trial. Lancet Oncol..

[120] Li D., Liu S., Cheng C., Xu L., Zhao P. (2023). Efficacy and safety of transarterial chemoembolization plus lenvatinib in the treatment of advanced hepatocellular carcinoma: A meta-analysis. Medicine.

[121] Qian H., Yang L., Zhao W., Chen H., He S. (2018). A comparison of CD105 and CD31 expression in tumor vessels of hepatocellular carcinoma by tissue microarray and flow cytometry. Exp. Ther. Med..

[122] Mohamed A., Caltharp S.A., Wang J., Cohen C., Farris A.B. (2013). Hepatocellular Carcinoma Microvessel Density Quantitation with Image Analysis: Correlation with Prognosis. J. Anal. Oncol..

[123] Huang C.-W., Lin S.-E., Huang S.-F., Yu M.-C., Tang J.-H., Tsai C.-N., Hsu H.-Y. (2022). The Vessels That Encapsulate Tumor Clusters (VETC) Pattern Is a Poor Prognosis Factor in Patients with Hepatocellular Carcinoma: An Analysis of Microvessel Density. Cancers.

[124] National Comprehensive Cancer Network NCCN Clinical Practice Guidelines in Oncology: Hepatocellular Carcinoma. Version 1.2025. https://www.nccn.org/guidelines/guidelines-detail?category=1&id=1460.

[125] Vogel A., Chan S.L., Dawson L.A., Kelley R.K., Llovet J.M., Meyer T., Ricke J., Rimassa L., Sapisochin G., Vilgrain V. (2025). Hepatocellular carcinoma: ESMO Clinical Practice Guideline for diagnosis, treatment and follow-up☆. Ann. Oncol..

[126] Han Y., Liu D., Li L. (2020). PD-1/PD-L1 pathway: Current researches in cancer. Am. J. Cancer Res..

[127] Li Q., Han J., Yang Y., Chen Y. (2022). PD-1/PD-L1 checkpoint inhibitors in advanced hepatocellular carcinoma immunotherapy. Front. Immunol..

[128] Hao L., Li S., Deng J., Li N., Yu F., Jiang Z., Zhang J., Shi X., Hu X. (2023). The current status and future of PD-L1 in liver cancer. Front. Immunol..

[129] Grywalska E., Pasiarski M., Góźdź S., Roliński J. (2018). Immune-checkpoint inhibitors for combating T-cell dysfunction in cancer. Onco Targets Ther..

[130] Sharpe A.H., Pauken K.E. (2018). The diverse functions of the PD1 inhibitory pathway. Nat. Rev. Immunol..

[131] Finn R.S., Ryoo B.-Y., Merle P., Kudo M., Bouattour M., Lim H.Y., Breder V., Edeline J., Chao Y., Ogasawara S. (2020). Pembrolizumab As Second-Line Therapy in Patients with Advanced Hepatocellular Carcinoma in KEYNOTE-240: A Randomized, Double-Blind, Phase III Trial. J. Clin. Oncol..

[132] Zhu A.X., Finn R.S., Edeline J., Cattan S., Ogasawara S., Palmer D., Verslype C., Zagonel V., Fartoux L., Vogel A. (2018). Pembrolizumab in patients with advanced hepatocellular carcinoma previously treated with sorafenib (KEYNOTE-224): A non-randomised, open-label phase 2 trial. Lancet Oncol..

[133] Lu L.-C., Lee Y.-H., Liu T., Shao Y.-Y., Cheng A.-L., Hsu C.-H. (2024). Abstract 1526: The distinct tumor microenvironment of advanced hepatocellular carcinoma with high PVR or high PD-L1 expression. Cancer Res..

[134] Li J.-H., Ma W.-J., Wang G.-G., Jiang X., Chen X., Wu L., Liu Z.-S., Zeng X.-T., Zhou F.-L., Yuan Y.-F. (2018). Clinicopathologic Significance and Prognostic Value of Programmed Cell Death Ligand 1 (PD-L1) in Patients with Hepatocellular Carcinoma: A Meta-Analysis. Front. Immunol..

[135] Sideras K., de Man R.A., Harrington S.M., Polak W.G., Zhou G., Schutz H.M., Pedroza-Gonzalez A., Biermann K., Mancham S., Hansen B.E. (2019). Circulating levels of PD-L1 and Galectin-9 are associated with patient survival in surgically treated Hepatocellular Carcinoma independent of their intra-tumoral expression levels. Sci. Rep..

[136] Huang Y., Yu W. (2024). Advances in Immune Checkpoint Therapy in Hepatocellular Carcinoma. Br. J. Hosp. Med..

[137] Zeng Z., Yang B., Liao Z.-Y. (2020). Current progress and prospect of immune checkpoint inhibitors in hepatocellular carcinoma (Review). Oncol. Lett..

[138] Seidel J.A., Otsuka A., Kabashima K. (2018). Anti-PD-1 and Anti-CTLA-4 Therapies in Cancer: Mechanisms of Action, Efficacy, and Limitations. Front. Oncol..

[139] Pinato D.J., Guerra N., Fessas P., Murphy R., Mineo T., Mauri F.A., Mukherjee S.K., Thursz M., Wong C.N., Sharma R. (2020). Immune-based therapies for hepatocellular carcinoma. Oncogene.

[140] El-Rebey H.S., Abdou A.G., Sultan M.M., Ibrahim S.H., Holah N.S. (2021). The Profile and Role of Tumor-infiltrating Lymphocytes in Hepatocellular Carcinoma: An Immunohistochemical Study. Appl. Immunohistochem. Mol. Morphol..

[141] Wei H., Dong C., Li X. (2024). Treatment Options for Hepatocellular Carcinoma Using Immunotherapy: Present and Future. J. Clin. Transl. Hepatol..

[142] Liu H.-T., Jiang M.-J., Deng Z.-J., Li L., Huang J.-L., Liu Z.-X., Li L.-Q., Zhong J.-H. (2021). Immune Checkpoint Inhibitors in Hepatocellular Carcinoma: Current Progresses and Challenges. Front. Oncol..

[143] Chan L.L., Kwong T.T., Yau J.C.W., Chan S.L. (2025). Treatment for hepatocellular carcinoma after immunotherapy. Ann. Hepatol..

[144] Alden S.L., Lim M., Kao C., Shu D., Singal A.G., Noonan A., Griffith P., Baretti M., Ho W.J., Kamel I. (2023). Salvage Ipilimumab plus Nivolumab after Anti-PD-1/PD-L1 Therapy in Advanced Hepatocellular Carcinoma. Cancer Res. Commun..

[145] Han S., Chok A.Y., Peh D.Y.Y., Ho J.Z.-M., Tan E.K.W., Koo S.-L., Tan I.B.-H., Ong J.C.-A. (2022). The distinct clinical trajectory, metastatic sites, and immunobiology of microsatellite-instability-high cancers. Front. Genet..

[146] Mukai S., Kanzaki H., Ogasawara S., Ishino T., Ogawa K., Nakagawa M., Fujiwara K., Unozawa H., Iwanaga T., Sakuma T. (2021). Exploring microsatellite instability in patients with advanced hepatocellular carcinoma and its tumor microenvironment. JGH Open.

[147] Mulet-Margalef N., Linares J., Badia-Ramentol J., Jimeno M., Sanz Monte C., Manzano Mozo J.L., Calon A. (2023). Challenges and Therapeutic Opportunities in the dMMR/MSI-H Colorectal Cancer Landscape. Cancers.

[148] Taherifard E., Tran K., Saeed A., Yasin J.A., Saeed A. (2024). Biomarkers for Immunotherapy Efficacy in Advanced Hepatocellular Carcinoma: A Comprehensive Review. Diagnostics.

[149] Eso Y., Shimizu T., Takeda H., Takai A., Marusawa H. (2020). Microsatellite instability and immune checkpoint inhibitors: Toward precision medicine against gastrointestinal and hepatobiliary cancers. J. Gastroenterol..

[150] Lou E., Baca Y., Walker P., Shields A.F., Prakash A., Weinberg B.A., Saeed A., Goel S., Nabhan C., Korn W.M. (2023). Beyond CPS for PD-L1 scoring: Genetic alterations that impact efficacy of immunotherapy in hepatocellular carcinoma (HCC). J. Clin. Oncol..

[151] Rüschoff J., Schildhaus H.-U., Rüschoff J.H., Jöhrens K., Bocker Edmonston T., Dietmaier W., Bläker H., Baretton G., Horst D., Dietel M. (2023). Testing for deficient mismatch repair and microsatellite instability. Pathologie.

[152] Ntellas P., Chau I. (2024). Updates on Systemic Therapy for Hepatocellular Carcinoma. Am. Soc. Clin. Oncol. Educ. Book.

[153] Liu W.-R., Tian M.-X., Tang Z., Fang Y., Zhou Y.-F., Song S.-S., Jiang X.-F., Wang H., Tao C.-Y., Zhou P.-Y. (2020). Nine-factor-based immunohistochemistry classifier predicts recurrence for early-stage hepatocellular carcinoma after curative resection. Br. J. Cancer.

[154] Liu X., Jiang D., Liu Y., Xie K., Zhao Y., Liu F. (2024). Crispr-Cas9-based long non-coding RNA interference and activation identified that the aberrant expression of Myc-regulated ST8SIA6 antisense RNA 1 promotes tumorigenesis and metastasis in hepatocellular carcinoma. Cytojournal.

[155] Zhou L., Zhang X., Zhang C., Wang Y., Zhang J., Wang Y., Sui Y. (2024). Deciphering the synergistic role of tyrosyl-tRNA synthetase 1 and yes-associated protein 1: Catalysts of malignant progression in hepatocellular carcinoma. CytoJournal.

[156] Bai Y., Cui G., Sun X., Wei M., Liu Y., Guo J., Yang Y. (2024). Effect of Deletion of ANGPTL4 Gene on Viability, Migration and Invasion Ability and Apoptosis of Hepatocellular Carcinoma Cells. Discov. Med..

[157] Hoda R.S., Brogi E., D’Alfonso T.M., Grabenstetter A., Giri D., Hanna M.G., Kuba M.G., Murray M.P., Vallejo C.E., Zhang H. (2021). Interobserver Variation of PD-L1 SP142 Immunohistochemistry Interpretation in Breast Carcinoma: A Study of 79 Cases Using Whole Slide Imaging. Arch. Pathol. Lab. Med..

[158] KITAYA K., YASUO T. (2013). Inter-observer and intra-observer variability in immunohistochemical detection of endometrial stromal plasmacytes in chronic endometritis. Exp. Ther. Med..

[159] Goldstein N.S., Hewitt S.M., Taylor C.R., Yaziji H., Hicks D.G., MD Members of Ad-Hoc Committee on Immunohistochemistry Standardization (2007). Recommendations for Improved Standardization of Immunohistochemistry. Appl. Immunohistochem. Mol. Morphol..

[160] Patel K., Strother R.M., Ndiangui F., Chumba D., Jacobson W., Dodson C., Resnic M.B., Strate R.W., Smith J.W. (2016). Development of immunohistochemistry services for cancer care in western Kenya: Implications for low- and middle-income countries. Afr. J. Lab. Med..

[161] Sanchez J.A., Portillo S., Zarka M.A., Snedden D., Pyle D., Goodman H., Hayes D.F. (2021). Improving Oncology-Pathology Collaboration in Resource-Limited Settings: An American Society of Clinical Oncology/College of American Pathologists Initiative. Am. Soc. Clin. Oncol. Educ. Book.

[162] McGenity C., Clarke E.L., Jennings C., Matthews G., Cartlidge C., Freduah-Agyemang H., Stocken D.D., Treanor D. (2024). Artificial intelligence in digital pathology: A systematic review and meta-analysis of diagnostic test accuracy. Npj Digit. Med..

[163] Ercan C., Renne S.L., Di Tommaso L., Ng C.K.Y., Piscuoglio S., Terracciano L.M. (2024). Hepatocellular Carcinoma Immune Microenvironment Analysis: A Comprehensive Assessment with Computational and Classical Pathology. Clin. Cancer Res..

[164] Mostafa G., Mahmoud H., Abd El-Hafeez T., E.ElAraby M. (2024). The power of deep learning in simplifying feature selection for hepatocellular carcinoma: A review. BMC Med. Inform. Decis. Mak..

[165] Li J., Dong J., Huang S., Li X., Jiang J., Fan X., Zhang Y. Virtual Immunohistochemistry Staining for Histological Images Assisted by Weakly-supervised Learning. Proceedings of the 2024 IEEE/CVF Conference on Computer Vision and Pattern Recognition (CVPR).

[166] Blom S., Paavolainen L., Bychkov D., Turkki R., Mäki-Teeri P., Hemmes A., Välimäki K., Lundin J., Kallioniemi O., Pellinen T. (2017). Systems pathology by multiplexed immunohistochemistry and whole-slide digital image analysis. Sci. Rep..

[167] Harms P.W., Frankel T.L., Moutafi M., Rao A., Rimm D.L., Taube J.M., Thomas D., Chan M.P., Pantanowitz L. (2023). Multiplex Immunohistochemistry and Immunofluorescence: A Practical Update for Pathologists. Mod. Pathol..

